# Optoelectronic and Excitonic Study of XI_2_ (X = Si, Ge, Sn, and Pb) Monolayers Envisaging Potential Technological Applications

**DOI:** 10.1021/acsomega.5c08479

**Published:** 2025-11-28

**Authors:** Bill Darwin Aparicio-Huacarpuma, José Artigas dos Santos Laranjeira, Kleuton Antunes Lopes Lima, Elie Albert Moujaes, Alysson Martins Almeida Silva, Julio Ricardo Sambrano, Alexandre Cavalheiro Dias, Luiz Antônio Ribeiro Júnior

**Affiliations:** † Institute of Physics, University of Brasília, Brasília, DF 70919-970, Brazil; ‡ Computational Materials Laboratory, LCCMat, Institute of Physics, University of Brasília, Brasília, DF 70919-970, Brazil; § Modeling and Molecular Simulation Group, School of Sciences, 28108 São Paulo State University (UNESP), Bauru, SP 17033-360, Brazil; ∥ Department of Applied Physics and Center for Computational Engineering and Sciences, State University of Campinas, Campinas, SP 13083-859, Brazil; ⊥ Institute of Physics, Federal University of Bahia, Campus Ondina, Salvador 40170-115, Brazil; # Department of Mechanical Engineering, College of Technology, 564113 University of Brasília, Brasilia 70910-900, Brazil; ∇ Institute of Physics and International Center of Physics, University of Brasília, Brasília, DF 70919-970, Brazil

## Abstract

This study investigates the structural stability and electronic, mechanical, excitonic, and optical properties of 2D-XI_2_ (X = Si, Ge, Sn, Pb) monolayers using both first-principles and semiempirical calculations. Our findings reveal that this group has semiconductor characteristics with band gaps from 2.35 to 3.28 eV at the HSE06 level. The excitonic effects are significant with exciton binding energies between 372 and 422 meV. They present a maximum solar harvesting efficiency, at the Shockley–Queisser limit, considering an electron–hole coupling of 16.37%. These findings indicate that these structures are promising for future optoelectronic applications, showing excellent visible and ultraviolet response and enhancing the photovoltaic cell performance.

## Graphical abstract

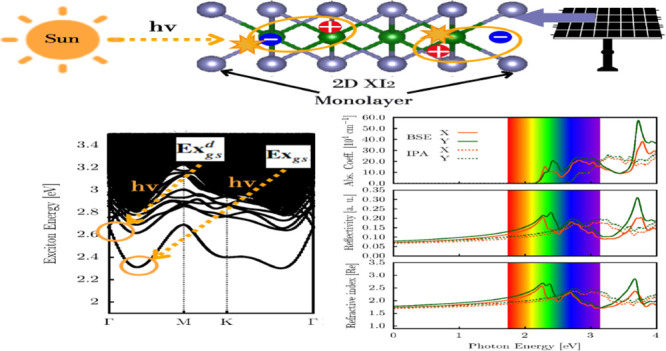

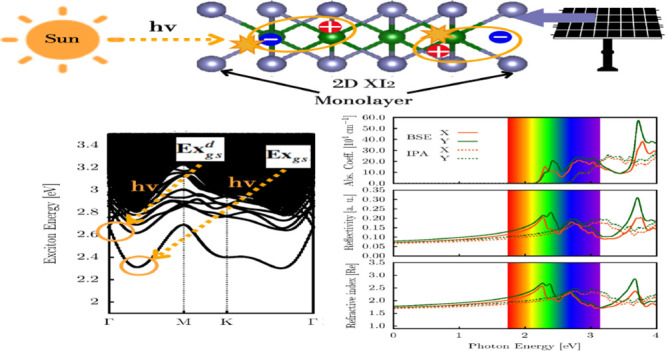

## 1 Introduction

In the past two decades, advances in synthesizing two-dimensional (2D) materials have led to theoretical and experimental studies of promising candidates for diverse applications. Notable examples include 2D carbon materials,^1–3^ MXenes,^4–6^ TMDs,^7–10^ h-BN,^11,12^ 2D perovskites,^13–16^ trichalcogenides,^17^ and 2D metal oxides.^18^ In this way, recently, 2D group-IV diiodide materials have attracted attention.^19–24^

In 2017, Zhong et al. successfully fabricated PbI_2_ monolayers using physical vapor deposition (PVD) and identified it as an indirect bandgap semiconductor.^25^ After 1 year, Liu et al. theoretically studied the GeI_2_ nanosheet, identifying it as an intrinsic semiconductor with a 2.59 eV bandgap energy.^26^ In 2019, Hoat et al.^27^ investigated how strain and external electric fields affect the electronic structure of the GeI_2_ monolayer, identifying that the band gap of the GeI_2_ monolayer can be effectively tuned by strain, showing a sharp decrease under compressive biaxial strain beyond – 6% and a nearly linear increase under uniaxial strain, while only strong external electric fields (≥0.6 eV/Å/e) significantly reduce the band gap. Optoelectronic properties of the PbI_2_ monolayer under uniaxial strain from first-principles calculations were also reported.^28^ Yuan et al. synthesized and characterized an SnI_2_ monolayer with a 2.9 eV indirect band gap using molecular beam epitaxy, corroborated by density functional theory (DFT) calculations.^29^

Additionally, DFT combined with the Boltzmann transport theory calculations revealed that monolayers of SnI_2_ and SiI_2_ exhibit a strong thermoelectric performance at low temperatures, achieving a maximum ZT value of 0.83 at 600 K. Both materials possess indirect band gaps of 2.06 and 1.63 eV, respectively, and display strong ultraviolet (UV) absorption, making them promising candidates for thermoelectric energy conversion and UV photodetector applications.^30^ Bolen et al.^31^ investigated monolayer PbI_2_ and reported ultralow thermal conductivity (0.052 W/mK), attributed to its low Debye temperature (69 K), heavy atomic mass, and strong phonon–phonon scattering (γ = 1.37). These features make the material a promising candidate for thermoelectric and thermal insulation applications.

Despite the promising results discussed above, which underscore the high potential of these materials for applications in optoelectronics, thermoelectrics, and related technologies, a detailed investigation of their optical and excitonic properties remains lacking in the literature. To address this gap, we systematically explore the structural, electronic, and optical properties of 2D-XI_2_ diiodide monolayers, with particular emphasis on their excitonic effects. Our analysis combines first-principles calculations within the DFT framework and a semiempirical tight-binding model based on maximally localized Wannier functions (MLWF-TB). Furthermore, the photovoltaic performance was assessed using both the independent particle approximation (IPA) and the Bethe–Salpeter equation (BSE), within the frameworks of the spectroscopy-limited maximum efficiency (SLME) and the Shockley–Queisser (SQ) limit.

## 2 Computational Details

The calculations were performed using the Vienna Ab initio Simulation Package (VASP),^32,33^ within the DFT framework^34,35^ and employing the projector-augmented wave (PAW) method.^36,37^ We utilized the Perdew–Burke–Ernzerhof (PBE) functional^38,39^ to evaluate the exchange and correlation energies for structural optimization, phonon spectra, and electronic properties. A high-energy cutoff of 700 eV was set to ensure the numerical accuracy. The Brillouin zone was sampled with dense *k*-point grids of 15 × 15 × 1 and 30 × 30 × 1, respectively, for the electronic band structure and density of states calculations for XI_2_. A vacuum layer of 20 Å was introduced along the *z*-axis to eliminate spurious interactions between the periodic images. The convergence criterion was set to 10^–6^ eV for the total energy and 0.01 eV/Å for the residual forces. The hybrid exchange-correlation Heyd–Scuseria–Ernzerhof (HSE06) hybrid functional^40^ was applied to obtain the band structures to obtain precise values of the band gaps.^41,42^

We examined the dynamical stability through a phonon dispersion analysis of each structure, as facilitated by the Phonopy package.^43^ This analysis was carried out by applying the density functional perturbation theory (DFPT) in conjunction with a 2 × 2 × 1 supercell, maintaining the same **k**-point density employed in the electronic band structure calculations. MLWFs were also constructed using the Wannier90 package^44^ to parametrize a TB Hamiltonian derived from DFT calculations with the HSE06 functional.

We also analyzed the thermodynamic stability through ab initio molecular dynamics (AIMD) simulations using the FHI-aims code,^45,46^ utilizing numerical atom-centered orbitals (NAOs) light the first tier basis set. All simulations was performed at the NVT ensemble with the Nosé–Hoover thermostat, at 300 K, during 5 ps with a time step of 1 fs, with a 3 × 3 × 1 supercell using the same **k**-points density as the previous calculations.

The excitonic and optical properties were calculated using the WanTiBEXOS code.^47^ The calculations included *s*–*p* orbital projections for the Si, Ge, Sn, Pb, and I atoms. Optical properties were evaluated using the IPA, which excludes excitonic effects, and by using the BSE^48^ approach, which incorporates electron–hole interactions. For these calculations, a **k**-point density of 120 AA^–1^ in the 2D plane was employed. The BSE calculations employed a 2D truncated Coulomb potential (V2DT)^49^ to account for the effects of reduced dimensionality. The dielectric functions were computed using a smearing factor of 0.05 eV, with a focus on the lowest conduction band and the three highest valence bands to simulate the optical and excitonic properties.

The power conversion efficiency (PCE) of the materials was calculated using the WanTiBEXOS code, which exploited both the SQ limit^50^ and the spectral loss model for efficiency (SLME) approach.^51^ Unlike the SQ limit, which requires only the band gap (or the exciton ground state when considering quasiparticle effects), the SLME approach takes into account whether the band gaps are direct or indirect as well as the optical absorption coefficient. The AM1.5G solar spectrum model^52,53^ was employed, assuming an operating temperature of 300 K. For more details, see the Supporting Information, Sections S3, S4, and S5.

## 3 Results and Discussion

### 3.1 Structural and Stability Properties

Figure 1a,b displays the top and side views of the 2D-XI_2_ monolayers, as visualized using VESTA software.^54^ These materials adopt a trigonal structure belonging to the *P*3̅*m*1 space group (1T phase), characterized by lattice angles of α = β = 90° and γ = 120°. Each monolayer comprises three atomic layers arranged in an I–X–I configuration within the *xy* plane, where the central atom (X = Si, Ge, Sn, or Pb) is sandwiched between two iodine­(I) atoms. Green spheres represent X atoms, while gray spheres denote iodine atoms. Four different compositions were considered in this work: SiI_2_, GeI_2_, SnI_2_, and PbI_2_.

**Figure 1:**
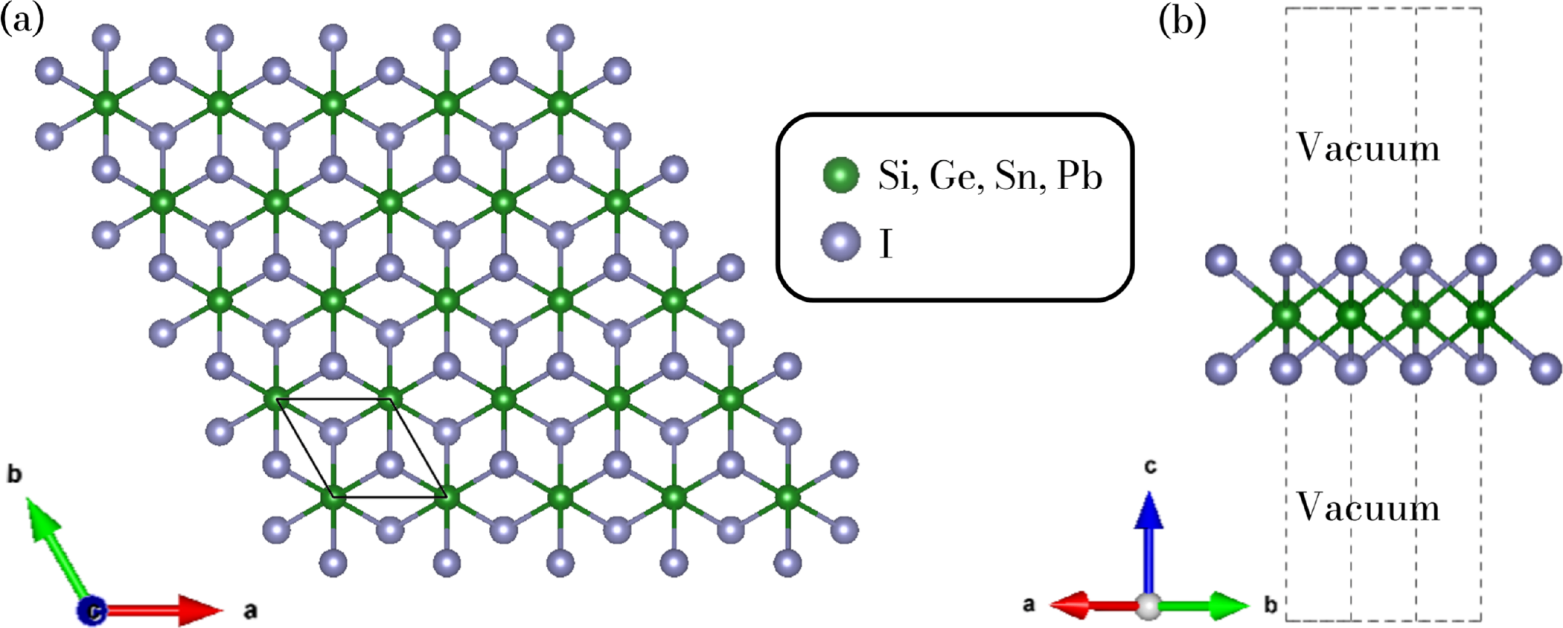
Top (a) and side (b) views of the geometry of the trigonal 2D-XI_2_ monolayers.

The optimized lattice parameters (*a*_0_) of the 2D-XI_2_ monolayers are summarized in Table 1, following the trend PbI_2_ (4.640 Å) > SnI_2_ (4.544 Å) > GeI_2_ (4.242 Å) > SiI_2_ (4.176 Å). This sequence is consistent with the atomic radii of the X elements—Pb (2.49 Å), Sn (2.48 Å), Ge (2.34 Å), and Si (2.32 Å).^55^ The calculated values show excellent agreement with previously reported results, with a relative deviation of approximately 0.1%.^24,27,28,30^ Moreover, a direct correlation is observed between the in-plane lattice parameter and the monolayer thickness (*t*_0_), which varies from 6.754 to 7.078 Å. The cohesive energy of the hexagonal XI_2_ (X = Si, Ge, Sn, and Pb) is first calculated to assess their thermodynamic stability, as defined by the formula, $E_{\mathrm{coh}}=\frac{E_{{\mathrm{XI}}_{2}}-E_{X}-2E_{I}}{3}$ , where *E*_XI_2__, *E*_X_, and *E*_I_ represent the total energies of XI_2_ monolayers, and isolated X (X = Si, Ge, Sn, Pb), and I atoms, respectively.^56^

**Table 1:** Cohesive Energy (*E*_coh_), Lattice Parameter (*a*_0_), and Monolayer Thickness, Including the van der Waals Length Correction of 3.3 Å (*t*_0_) for XI_2_ Monolayers

system	*E*_coh_ (eV/atom)	*a*_0_ (Å)	*t*_0_ (Å)
SiI_2_	–2.456	4.176	6.754
GeI_2_	–2.452	4.242	6.870
SnI_2_	–2.457	4.544	7.051
PbI_2_	–2.522	4.640	7.078

The cohesive energy (*E*_coh_) of all 2D-XI_2_ monolayers has negative values. Specifically, the SiI_2_ structure exhibits a cohesive energy of −2.456 eV/atom, while GeI_2_ presents a similar value of −2.452 eV/atom. The SnI_2_ monolayer shows a slightly higher value of −2.457 eV/atom, and PbI_2_ demonstrates the lowest cohesive energy among the four at −2.522 eV/atom. These results indicate that the arrangement of this monolayer is energetically favorable.

Figure 2 presents phonon dispersion calculations, indicating dynamic stability. Figure 2a–d depicts positive frequencies, confirming the stability of all monolayers. The phonon analysis shows three acoustic and six optical modes with vibrational modes below 9 THz (300 cm^–1^) for SiI_2_, 6 THz (200 cm^–1^) for GeI_2_, 5 THz (166 cm^–1^) for SnI_2_, and 4 THz (133 cm^–1^) for PbI_2_. In SiI_2_, GeI_2_, and SnI_2_, the absence of gaps between acoustic and optical modes suggests enhanced phonon scattering, influencing thermal transport properties.^56,57^ However, PbI_2_ shows a small gap, as seen in Figure 2d.

**Figure 2:**
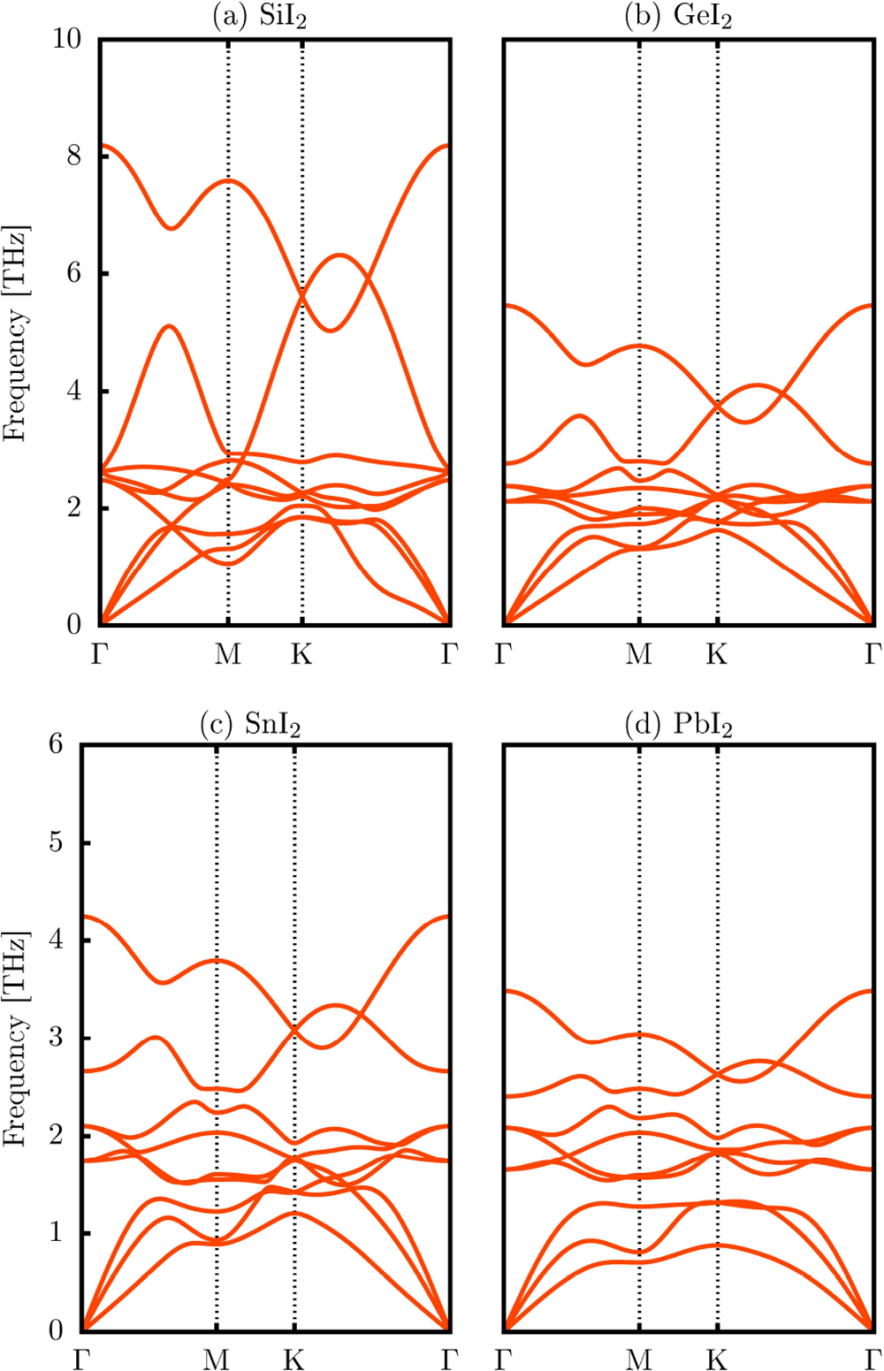
Phonon dispersion of the XI_2_ monolayers (X = Si, Ge, Sn, or Pb) calculated with the PBE functional.

In addition, thermodynamic properties, such as the Helmholtz free energy, entropy, and heat capacity as a function of temperature in K were obtained. All trends as a function of temperature are plotted in Figures S1–S4 in the Supporting Information, where these four monolayers present negative values of the Helmholtz free energy for temperatures beyond 100 K.

The mechanical and elastic properties of all of the studied monolayers were systematically evaluated. Polar plots of the Young’s modulus, shear modulus, and Poisson’s ratio, derived from the elastic constants *C*_*ij*_, are presented in Figure 3. Given the hexagonal symmetry of these 2D layered systems, four in-plane elastic constants are required; *C*_11_, *C*_22_, *C*_12_, and *C*_66_, such that *C*_11_ = *C*_22_.

**Figure 3:**
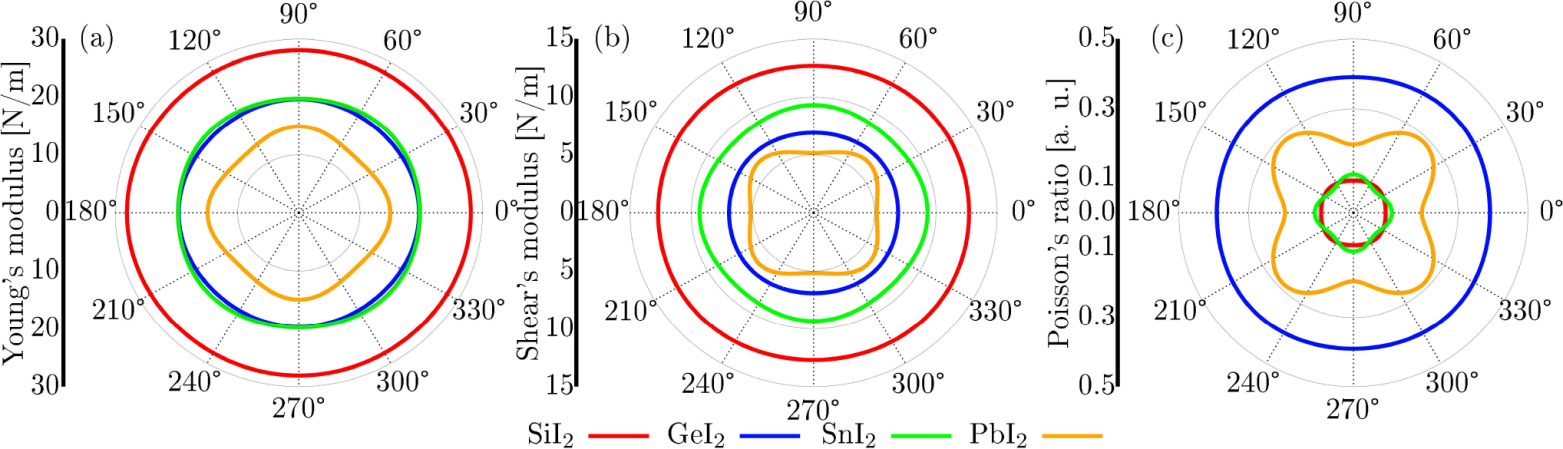
Polar plots of (a) Young’s modulus *Y* (θ), (b) shear modulus *G*(θ), and (c) Poisson’s ratio ν­(θ) for the XI_2_ monolayers.

The calculated elastic constants, along with the maximum values of Young’s modulus (*Y*), shear modulus (*G*), and Poisson’s ratio (ν = *C*_12_/*C*_11_), are summarized in Table 2. All values fulfill the Born–Huang stability criteria,^58^ namely, *C*_11_ = *C*_22_ > 0, *C*_66_ > 0, and *C*_11_^2^ > *C*_12_^2^, confirming that the SiI_2_, GeI_2_, SnI_2_, and PbI_2_ monolayers are mechanically stable.

**Table 2:** Elastic Constants *C*_*ij*_ (N/m), Maximum Young’s Modulus *Y*_max_ (N/m), Maximum Shear Modulus *G*_max_ (N/m), and Maximum Poisson’s Ratio ν_max_ for the XI_2_ Monolayers (X = Si, Ge, Sn, Pb)

system	*C*_11_ (N/m)	*C*_22_ (N/m)	*C*_12_ (N/m)	*C*_66_ (N/m)	*Y*_max_ (N/m)	*G*_max_ (N/m)	ν_max_
SiI_2_	28.33	28.33	2.62	12.70	28.08	12.85	0.10
GeI_2_	23.18	23.18	9.06	6.90	19.63	7.05	0.40
SnI_2_	19.99	19.99	2.20	9.11	20.25	9.31	0.11
PbI_2_	15.52	15.52	3.04	5.16	14.93	6.24	0.29

Figure 3a–c displays the polar plots of Young’s modulus, shear modulus, and Poisson’s ratio for the studied monolayers. Among them, SiI_2_ exhibits a nearly isotropic mechanical behavior, followed by PbI_2_, SnI_2_, and GeI_2_, which show increasing degrees of anisotropy. The maximum values of Young’s modulus range from 14.93 to 28.08 N/m, while the maximum shear modulus spans from 6.24 to 12.85 N/m, in agreement with previously reported results.^20,59^

Regarding Poisson’s ratio, the highest value of 0.40 is observed for the GeI_2_ monolayer, indicating a pronounced lateral deformation under uniaxial stress. In contrast, SiI_2_ presents the lowest value of 0.10, suggesting a higher resistance to transverse strain. Intermediate values are found for SnI_2_ (0.11) and PbI_2_ (0.29). These results are consistent with those observed in other theoretical studies on 2D monolayers.^6,56^

Among the systems analyzed, SiI_2_ exhibits the highest mechanical stiffness, as reflected by its maximum Young’s and shear moduli. In contrast, GeI_2_ stands out as the most flexible material due to its relatively lower elastic constants and elevated Poisson’s ratio.

While the hexagonal lattice symmetry dictates *C*_11_ = *C*_22_, perfect in-plane isotropy in the linear elastic regime further requires the condition 2*C*_66_ = *C*_11_ – *C*_12_. The degree to which this condition is met can be quantified by the 2D Zener anisotropy ratio,^60^ *A*_2D_ = 2*C*_66_/(*C*_11_ – *C*_12_), where *A*_2D_ = 1 indicates perfect isotropy. Our calculations yield *A*_2D_ values of 0.988 for SiI_2_, 0.977 for GeI_2_, 1.024 for SnI_2_, and 0.827 for PbI_2_. This confirms that SiI_2_, GeI_2_, and SnI_2_ are nearly isotropic, whereas PbI_2_ exhibits a moderate in-plane anisotropy. This physical origin of the anisotropy for PbI_2_ can be linked to its characteristically soft lattice, evident in its low elastic constants and phonon frequencies, which enhances its shear compliance and leads to a more pronounced reduction of *C*_66_ relative to the isotropic expectation.

To complement the structural stability analysis, we performed AIMD simulations to investigate the thermodynamic stability of these monolayers at 300 K, as shown in Figure 4. These results shows small total energy oscillations around an average value after thermalization in the first picosecond, where the system progressed from 0 to 300 K, showing that all monolayers are thermodynamically stable at 300 K. The detailed results from AIMD showing the total energy and temperature variations are shown in the Supporting Information Figures S5–S8.

**Figure 4:**
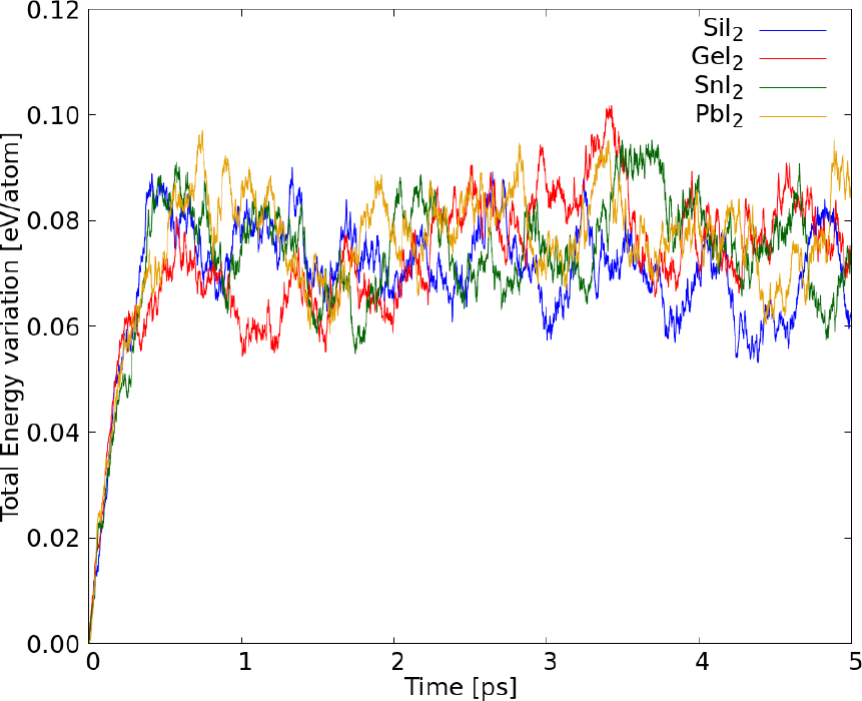
Total energy variation per atom in AIMD simulations of XI_2_ monolayers.

### 3.2 Electronic Properties

To investigate the electronic properties of the XI_2_ (X = Si, Ge, Sn, or Pb) monolayers, band structure calculations were performed using three approaches: the generalized gradient approximation with the PBE functional (black curve), PBE including spin–orbit coupling (PBE+SOC, yellow dashed curve), and the hybrid HSE06 functional (orange curve), as illustrated in Figure 5. All monolayers exhibit a semiconducting behavior with an indirect bandgap character, following the high-symmetry path Γ–M–K−Γ. In Figure 5a–d, the conduction band minimum (CBM) is located near the Γ point, while the valence band maximum (VBM) lies along the Γ–M or K−Γ segments, depending on the specific monolayer.

**Figure 5:**
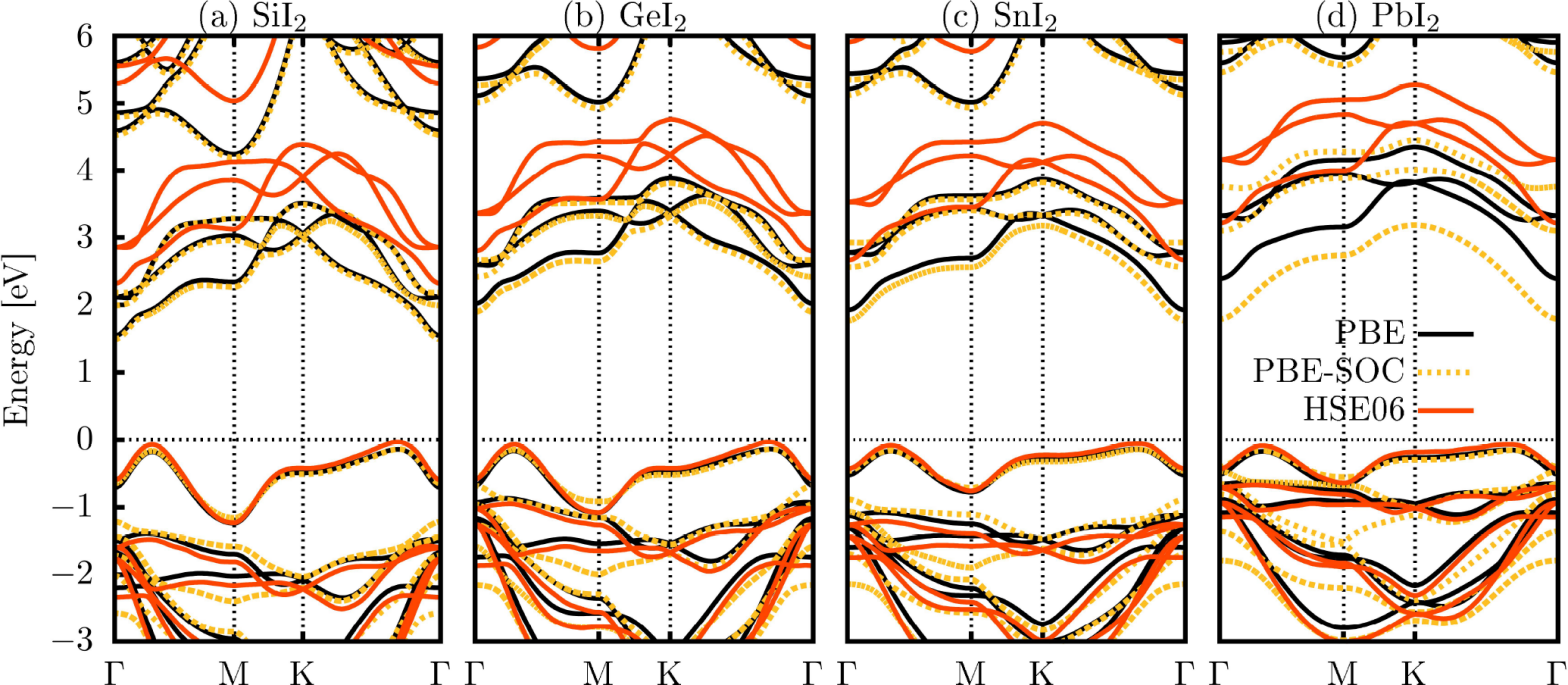
Electronic band structures of XI_2_ (X = Si, Ge, Sn, Pb) monolayers computed along the high-symmetry path Γ–M–K−Γ in the Brillouin zone using PBE (with and without spin–orbit coupling) and HSE06 functionals. The Fermi level is aligned with the valence band maximum.

[Table tbl3] Table 3 displays the band gaps obtained at the three different levels considered above. As can be noticed, PBE results align well with prior theoretical findings.^24,27,28,30^ As shown in Figure 5a–c, the SiI_2_, GeI_2_, and SnI_2_ monolayers have minimal band structure differences between PBE and PBE+SOC, indicating the low impact of the SOC term. In contrast, Figure 5d shows a larger bandgap variation in PbI_2_ when SOC is included. Due to the underestimation of the band gap using the PBE functional compared with experimental values,^41,61,62^ the HSE06 hybrid functional is believed to provide a more accurate approximation. Employing the HSE06 functional, bandgap energies of 2.39 2.86, 2.73, and 3.29 eV were obtained for SiI_2_, GeI_2_, SnI_2_, and PbI_2_ monolayers, respectively.

**Table 3:** Electronic Band Gap of PBE (eV), PBE+SOC (eV), and HSE06 (eV) Functional in Comparison with PBE Results of Published References of XI_2_ Monolayers

		*E*_g_ (eV)	
Syst.	PBE	PBE+SOC	HSE06
SiI_2_	1.68 (1.63, 1.67 ^24,30^ )	1.63	2.35
GeI_2_	2.15 (2.09, 2.19 ^24,27^ )	2.03	2.83
SnI_2_	2.06 (2.06, 2.03 ^24,30^ )	1.91	2.72
PbI_2_	2.54 (2.48, 2.50 ^24,28^ )	1.95	3.28

Figure 6a–d shows the total density of states (TDOS) and projected density of states (PDOS) for the SiI_2_, GeI_2_, SnI_2_, and PbI_2_ monolayers, respectively. In Figure 6a, the valence band (VB) of SiI_2_ is primarily composed of the *p* orbitals of iodine and the *s* orbitals of silicon, whereas the conduction band (CB) mainly involves contributions from the *p* orbitals of both iodine and silicon. For GeI_2_ (Figure 6b), the iodine *p* orbitals dominate both the VB and the CB, although the *p* orbitals of germanium become more prominent in the CB region. A similar pattern is observed for the SnI_2_ and PbI_2_ monolayers, as shown in Figure 6c,d, respectively, where the *p* orbitals of iodine and the respective group-IV element contribute significantly to the electronic states near the Fermi level.

**Figure 6:**
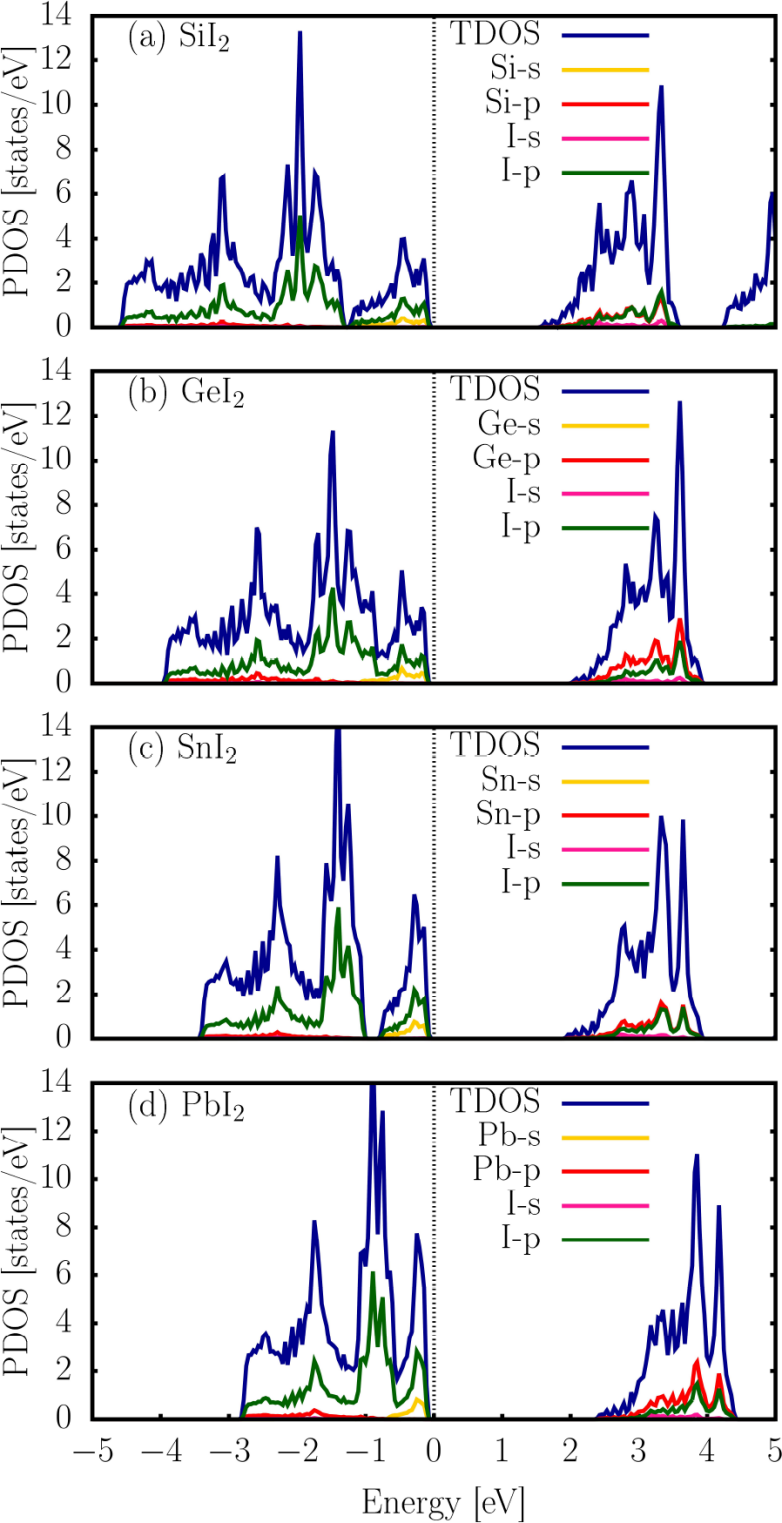
Projected density of states (PDOS) and total density of states (TDOS) of the SiI_2_, GeI_2_, SnI_2_, and PbI_2_ monolayers were calculated at the PBE level without spin–orbit coupling.

### 3.3 Excitonic and Optical Properties

We calculated the excitonic (quasiparticle) band energies of the aforementioned monolayers using the BSE formalism, which is implemented in the WantiBEXOS code.^47^ As illustrated in Figure 7a–d, the calculated exciton band structures reveal that the ground excitonic state, denoted as Ex_gs_, is always indirect. The most likely transition occurs along the high-symmetry path from K to Γ. This pattern aligns with the indirect nature of the electronic band gap, suggesting the involvement of phonon-assisted optical transitions. As a result, light-induced excitations can occur at energy levels lower than the direct optical band gap, which is typically determined by considering only momentum-conserving transitions, similar to observations reported for other 2D materials.^63^

**Figure 7:**
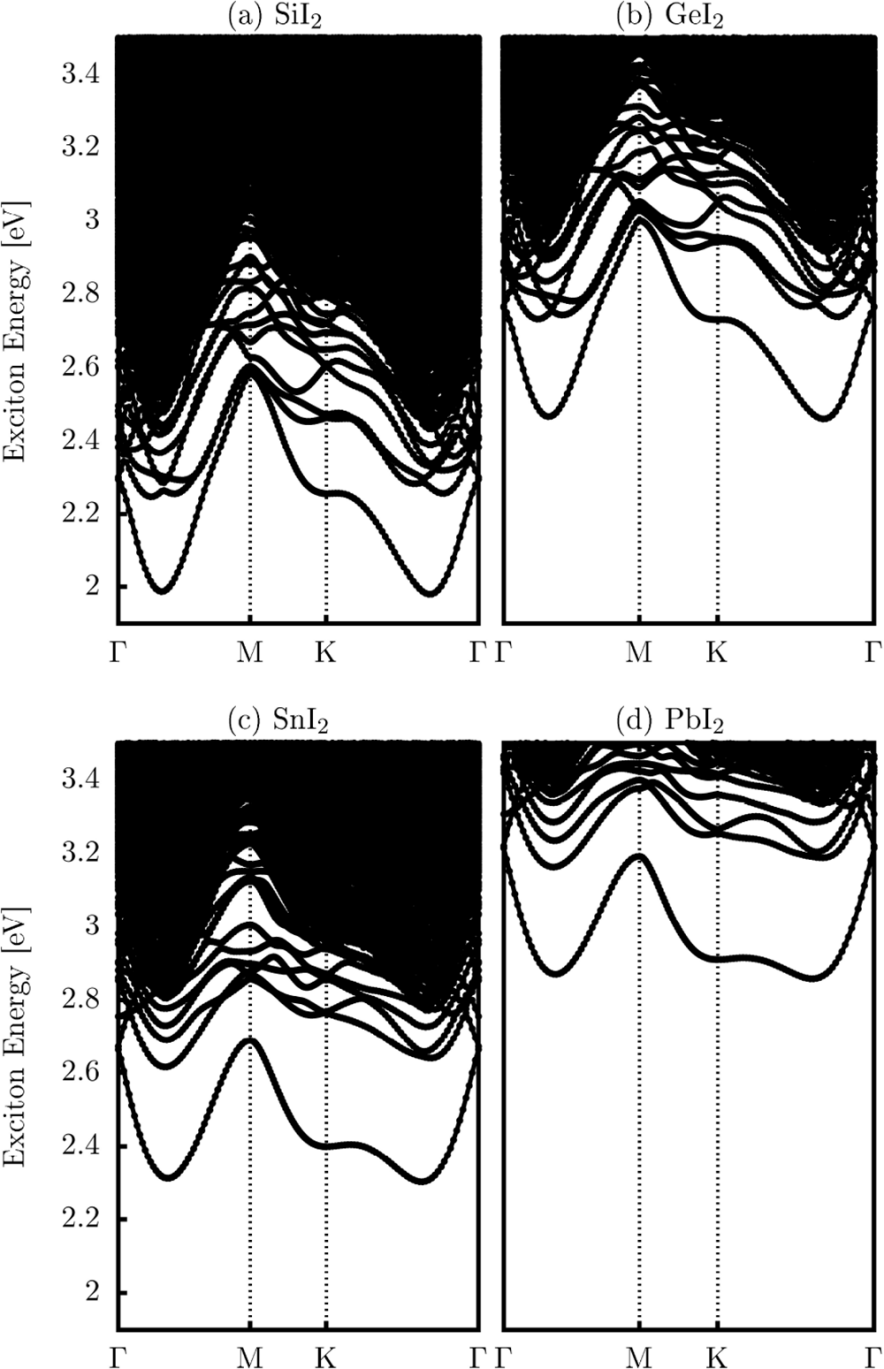
Excitonic band structures of the XI_2_ monolayers (X = Si, Ge, Sn, Pb) computed using the BSE approach. The excitonic states are resolved along the high-symmetry Γ–M–K−Γ path of the Brillouin zone.

As shown in Table 4, we report the fundamental band gap (*E*_g_), direct band gap (*E*_g_^d^), exciton ground-state energy (Ex_gs_), direct exciton ground state (Ex_gs_^d^), and exciton binding energy (Ex_b_) for the studied monolayers. The exciton binding energy quantifies the strength of quasiparticle interactions and plays a crucial role in determining the optical response in the linear regime of 2D semiconductors. The calculated Ex_b_ values for the SiI_2_, GeI_2_, SnI_2_, and PbI_2_ monolayers are 372, 373, 415, and 422 meV, respectively. These values are consistent with those reported for other 2D materials.^2,10,64,65^ The relatively large exciton binding energies observed in all cases indicate strong Coulomb quasiparticle interaction.

**Table 4:** Calculated MLWF-TB+BSE Excitonic Properties: Fundamental Band Gap (*E*_g_), Direct Band Gap (*E*_g_^d^), Exciton Ground State (Ex_gs_), Direct Exciton Ground State (Ex_gs_^d^), and Exciton Binding Energy (Ex_b_), Calculated as *E*_g_ – Ex_gs_

system	*E*_g_ (eV)	*E*_g_^d^ (eV)	Ex_gs_ (eV)	Ex_gs_^d^ (eV)	Ex_b_ (meV)
SiI_2_	2.35	2.75	1.98	2.29	372
GeI_2_	2.83	3.23	2.46	2.76	373
SnI_2_	2.72	3.09	2.30	2.66	415
PbI_2_	3.28	3.62	2.86	3.21	422

We further calculated the optical absorption coefficients in the *x* and *y* directions. These linear optical responses were obtained using two different computational approaches. The first approach is the IPA, which ignores electron–hole interactions. The second approach is the BSE method, which explicitly takes into account the Coulomb attraction between electrons and holes. Figure 8a–d presents the absorption spectra computed using the BSE (solid orange and green curves) and IPA (dashed orange and green curves) for the monolayers of SiI_2_, GeI_2_, SnI_2_, and PbI_2_.

**Figure 8:**
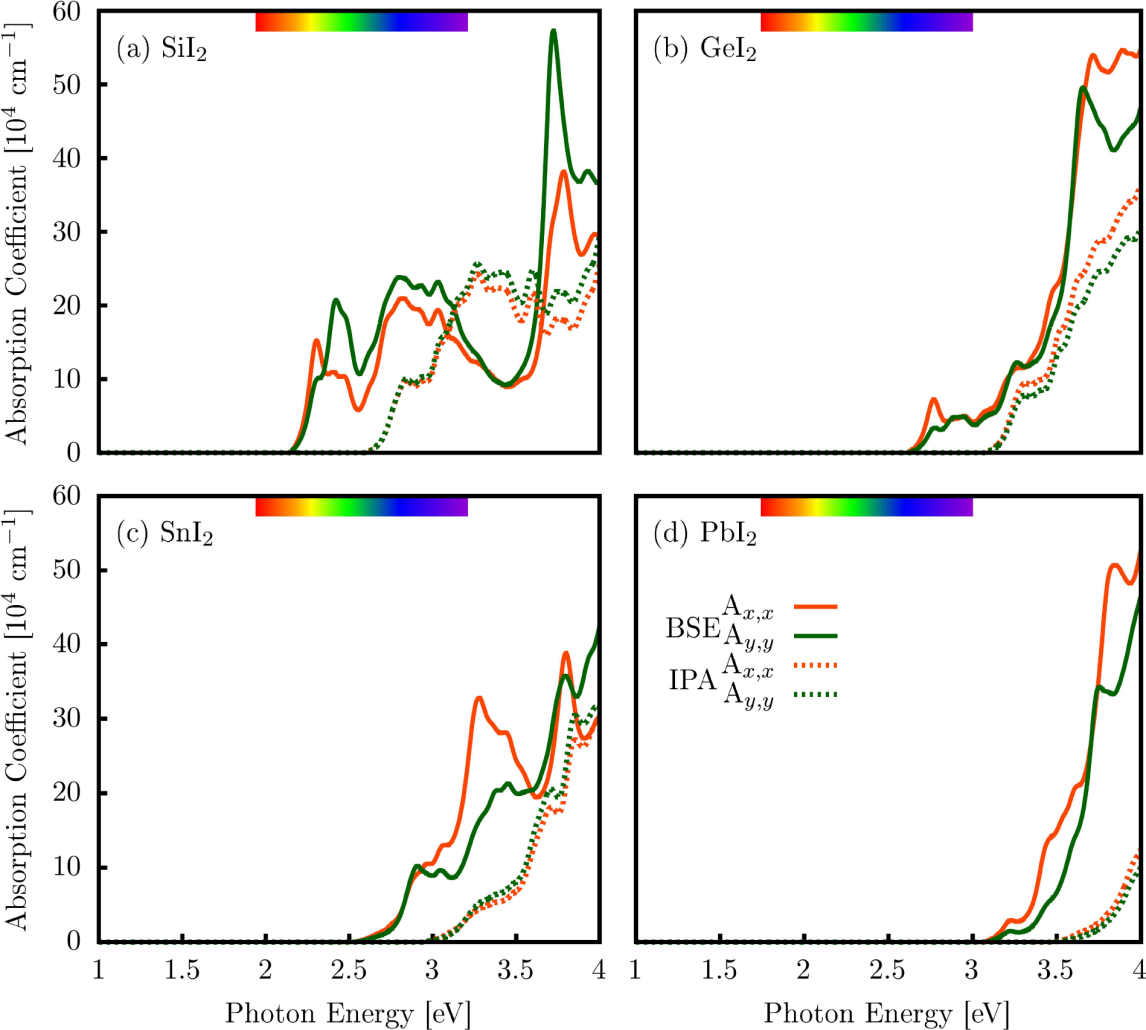
Comparison of the optical absorption coefficients for the XI_2_ (X = Si, Ge, Sn, Pb) monolayers calculated within the IPA and the BSE frameworks. The absorption spectra are resolved for linear light polarization along the *x* (orange curves) and *y* (green curves) directions.

The optical absorption coefficient plots for the SiI_2_ monolayer show notable peaks in the UV and visible regions, linked to the excitonic band gap for *x* and *y* light polarizations at the BSE level. In the *x* direction, the peaks occur at 2.31, 2.81, 3.03, and 3.78 eV, while in the *y* direction, they emerge at 2.29, 2.41, 2.80, 3.03, and 3.72 eV. The peak at 3.72 eV in the *y* polarization is the most significant, with an absorption coefficient of 57 × 10^4^ cm^–1^, as depicted in Figure 8a. In the UV region, the main absorption edge at 3.78 eV in the *x* direction is around 38 × 10^4^ cm^–1^. At the IPA level, the main absorption peaks are nearly isotropic in the UV region up to 25 × 10^4^ cm^–1^, occurring at 2.84 3.26, 3.43, and 3.60 eV in both directions.

Interestingly, the GeI_2_ monolayer shows no pronounced peaks at the IPA level; however, the absorption coefficient increases isotropically in both directions, reaching a maximum value of 30 × 10^4^ cm^–1^. At the BSE level, distinct peaks appear in the *x* direction of polarized light at 2.77, 3.47, 3.71, and 3.88 eV. In comparison, the *y* direction displays only two peaks, located at 3.25 and 3.65 eV. As illustrated in Figure 8b, the maximum absorption coefficients are approximately 54 × 10^4^ and 50 × 10^4^ cm^–1^ for the *x* and *y* polarization directions, respectively.

Figure 8c presents the absorption coefficient for the SnI_2_ monolayer. It reveals important BSE peaks at 2.96, 3.05, 3.28, and 3.80 eV along the *x*-direction for polarized light, with main absorption peaks of 33 × 10^4^ and 38 × 10^4^ cm^–1^. In the *y*-direction, peaks occur at 2.91, 3.04, 3.44, and 3.79 eV, with a maximum absorption coefficient of 42 × 10^4^ cm^–1^ around 4.0 eV. At the IPA level, there are no significant differences between the polarizations, indicating isotropic light–matter interactions, with a maximum absorption coefficient close to 30 × 10^4^ cm^–1^.

In Figure 8d, the PbI_2_ monolayer at the BSE level shows peaks at 3.22, 3.45, 3.60, and 3.84 eV for light polarization along the *x* direction with a maximum absorption of 50 × 10^4^ cm^–1^ and at 3.21 and 3.75 eV for light polarized along the *y* direction with a peak absorption near 44 × 10^4^ cm^–1^, manifesting in the blue and UV regions. An isotropic character is observed using the IPA method, with the absorption coefficient increasing above 3.5 eV.

Additionally, the reflectivity properties and refractive indices as a function of photon energy are presented in Figures S9–S12 in the Supporting Information. All monolayers show an increase in reflectivity and refractive indices until they reach a maximum value at an approximate energy of 3.80 eV.

In all four monolayers, we observe a red shift and the presence of more peaks when the BSE is applied, indicating the significance of electron–hole interactions. Notably, some monolayers respond to both the visible and UV regions, while others exhibit a response only in the UV region. This suggests that these monolayers may be suitable for a variety of applications including photodetectors, light sensors, and photovoltaic cells.

### 3.4 Insights into the Solar Harvesting Performance

In this section, we assess the PCE of SiI_2_, GeI_2_, SnI_2_, and PbI_2_ monolayers using both the IPA and BSE methods.^47^ The evaluation is carried out within two widely adopted theoretical frameworks for the photovoltaic performance: the SLME model^51^ and the SQ limit.^50^ Our analysis considers three distinct formulations of photovoltaic efficiency: (i) the practical SLME-based efficiency (PCE^SLME^), which incorporates the actual optical absorption spectrum and material thickness; (ii) the idealized maximum SLME efficiency (PCE_max_^SLME^), assuming a step-function absorption onset at the fundamental band gap; and (iii) the SQ limit efficiency (PCE^SQ^), which accounts solely for radiative recombination processes.^66^

To evaluate the PCE of the 2D-XI_2_ monolayers, we accounted for both direct and indirect electronic band gaps as well as the lowest-energy excitonic (ground-state) transitions. To estimate the theoretical upper bound of the PCE, we utilized the following relationships:

$$\mathrm{PCE}=\frac{J_{\mathrm{SC}}V_{\mathrm{OC}}}{P_{\mathrm{SOLAR}}}$$ 1

where *V*_OC_ is the open-circuit voltage, *J*_SC_ is the short-circuit current, and *P*_SOLAR_ is the total incident solar power per unit area. These are defined as follows:^10,63^

$$J_{\mathrm{SC}}=\int_{E_{g}^{d}}^{\infty }\frac{P(E)}{E}dE$$ 2

and

$$P_{\mathrm{SOLAR}}=\int_{0}^{\infty }P(E)dE$$ 3

In these expressions, *E*_g_^d^ corresponds to the electronic band gap of the donor material, and *P*(*E*) represents the AM1.5G solar spectral irradiance, commonly used as the reference spectrum for standard photovoltaic characterization under nonconcentrated sunlight conditions.^47,53^

The function *P*(*E*) accounts for atmospheric light absorption and scattering, constituting the conventional solar spectrum used for nonconcentrated photovoltaic conversion. The current density *J*(*V*) and voltage *V* determine the output power density. The maximum output power density, denoted as *P*_PV_, is obtained by maximizing the *J*–*V* characteristic of the illuminated solar cell, and is given by

$$P_{\mathrm{PV}}=J(V_{\max})V_{\max}$$ 4

where *V*_max_ is the voltage at which the maximum output power density is achieved. The current density *J*(*V*) is generally described by the following expression:

$$J(V)=J_{\mathrm{sc}}-\frac{j_{0}}{fr}\left(\exp\left(\frac{eV}{k_{B}T}\right)-1\right)$$ 5

*k*_B_ is the Boltzmann constant, *e* is the elementary charge, *fr* is the radiative electron–hole recombination fraction, *T* is the temperature of the solar cell, and *J*_sc_ is the short-circuit current density, also referred to as the illuminated or photogenerated current. The latter is calculated using the following relation:

$$J_{\mathrm{sc}}=e\int_{0}^{\infty }a(E)\frac{P(E)}{E}dE$$ 6

where *a*(*E*) denotes the absorbance, defined as the ratio of the absorbed power to the incident solar power at energy *E*. The reverse saturation current density, *J*_0_, is determined based on the principle of detailed balance under thermal equilibrium, assuming that the radiative recombination rate equals the photon absorption rate from the environment.

The model presumes that the solar cell is in thermal contact with a perfect heat sink, maintaining its temperature to be equal to that of the surroundings. As a result, the ambient radiation is described by the blackbody spectrum at temperature *T*:

$$J_{0}=e\pi \int_{0}^{\infty }a(E)\Phi_{\mathrm{bb}}(E)dE$$ 7

where the blackbody photon flux Φ_bb_(*E*) is given by

$$\Phi_{\mathrm{bb}}(E)=\frac{2E^{2}}{h^{3}v_{c}^{2}}(e^{E/k_{B}T}-1{)}^{-1}$$ 8

with *h* being Planck’s constant and *v*_c_ the speed of light in vacuum.

Table 5 summarizes the PCE results obtained using the IPA and BSE methods. The PCE values for the SiI_2_, GeI_2_, SnI_2_, and PbI_2_ monolayers, calculated using the PCE^SLME^ limit, are notably low. For both the IPA and BSE methods, the values are below 0.6%. This efficiency is significantly lower than the theoretical maximum predicted by the SQ limit. This discrepancy can be attributed to the ultrathin nature of these monolayers, which have thicknesses of approximately 6.75 6.87, 7.05, and 7.08 Å (see Table 1). The reduced thickness leads to limited optical absorption, meaning that only a small portion of the incident photon flux is effectively absorbed. This limitation poses a fundamental challenge for the practical implementation of 2D monolayers in photovoltaic applications.

**Table 5:** Maximum PCE Values Calculated at the IPA and BSE Levels for the XI_2_ (X = Si, Ge, Sn, or Pb) Monolayers^a^

	IPA			BSE		
system	PCE^SLME^	PCE_max_^SLME^	PCE^SQ^	PCE^SLME^	PCE_max_^SLME^	PCE^SQ^
SiI_2_	0.24	6.45	7.84	0.54	13.57	16.37
GeI_2_	0.09	2.24	2.63	0.19	6.61	7.64
SnI_2_	0.06	3.02	3.54	0.23	7.83	9.39
PbI_2_	0.01	0.72	0.81	0.08	2.35	2.71

a The table reports the PCE obtained via the SLME method (PCE^SLME^) (%), the idealized SLME assuming 100% phonon-assisted absorption onset at the band gap (PCE_max_^SLME^) (%), and the SQ limit (PCE^SQ^) (%) at room temperature (*T* = 300 K).

Jariwala et al.^67^ demonstrated that employing light-trapping techniques can boost the absorbance to nearly 100%. This leads to a significant enhancement in the solar energy harvesting performance. The theoretical maximum efficiency of solar-limiting materials (SLME), denoted as PCE_max_^SLME^, reflects ideal light absorption conditions that can be achieved through optimized device architecture. The values for this parameter are summarized in Table 5. At the IPA level, the PCE ranges from 0.72 to 6.45% at the IPA level and from 2.35 to 13.57% at the BSE level. Notably, these values are slightly lower than those predicted by the SQ limit.

On the other hand, the PCE^SQ^ approach results at IPA level for these monolayers are 7.84% for SiI_2_, 2.63% for GeI_2_, 3.54% for SnI_2_, and around 0.81% for PbI_2_. These low values can be justified since the IPA optical band gaps are much larger than the optimum region for solar harvesting, which is around 1.34 eV.^50,67^ However, when BSE is used, the optical band gaps exhibit a red shift in the optical properties, implying a better response. Using the BSE level, we obtain values of the order of 16.37% for SiI_2_, 7.63% for GeI_2_, 9.39% for SnI_2_, and 2.71 eV for PbI_2_. Other important physical parameter values can be found in Tables S2 and S3 of the Supporting Information.

Compared with other 2D materials with the BSE level and at the PCE^SQ^ limit, Pd-based materials were reported in 1O_T_ (1T) phases with values of 24.21%(28.95%) for PdS_2_, 16.35% (32.32%) for PdSe_2_, and 16.00% (32.65%) for PdSSe.^68^ Also, bimetal MXenes exhibited PCE_max_^SLME^ (PCE^SQ^) values of 27.70% (31.70%) for ScYCBr_2_, 28.28% (31.43%) for ScYCCl_2_, 22.69% (27.08%) for ScYCF_2_, 28.82% (32.28%) for ScYCH_2_, 16.48% (32.55%) for ScYCI_2_, and 16.82% (23.44%) for ScYC­(OH)_2_.^64^ In addition, SiS_2_ and SiSe_2_ monolayers were reported with values of 2.34% (3.02%) for SiS_2_ and 17.63% (21.25%) for SiSe_2_ at PCE_max_^SLME^ (PCE^*textSQ*^) levels.^69^ Such values are directly related to the optical gap band, which is close to the optimal value of 1.34 eV.

The XI_2_ monolayers, where X can be Si, Ge, Sn, or Pb, present a promising alternative for photovoltaic applications. Recent years have seen successful synthesis and characterization of these materials.^21,23,25,29^ In our study, we observed PCE values of up to 16.37% (with a value of 13.57% at the SQ (SLME_max_) limits at the BSE level). These PCE values are influenced by the optical band gap and vary with the metal counterparts of the diiodide materials.

## 4 Conclusions

This study emphasizes the promising potential of 2D-XI_2_ monolayers (where X = Si, Ge, Sn, Pb) for applications in solar cells, based on thorough first-principles and semiempirical analyses. These materials demonstrate thermodynamic and mechanical stability as well as semiconducting properties with suitable band gaps ranging from 1.68 to 2.54 eV (using the PBE method) and 2.35 to 3.28 eV (using the HSE06 method). These characteristics make them ideal for solar energy absorption. Additionally, their strong exciton binding energies (from 372 to 422 meV) and excellent optical responses in both the visible and UV regions further enhance their suitability for next-generation optoelectronic devices. Remarkably, the calculated PCE reaches a peak of 16.37%, which is under the SQ limit. This fact accentuates the efficiency and feasibility of integrating these monolayers into photovoltaic cells. Overall, the findings establish 2D-XI_2_ monolayers as highly promising alternatives for future high-performance solar energy technologies.

## Supplementary Material



## Notes

Detailed data os crystal structures at the POSCAR format, thermodynamic properties obtained from phonon calculations, detailed BSE calculation parameters, mathematical formalism for PCE calculation, and complementary data from PCE (PDF)
