# Supplementary material for: Optoelectronic and Excitonic Study of XI2 (X = Si, Ge, Sn, and Pb) Monolayers Envisaging Potential Technological Applications
Source: ACS Omega. 2025 Nov 28;10(48):59219–29. doi: 10.1021/acsomega.5c08479 (PMC12771178; doi:10.1021/acsomega.5c08479)
Supplement: Supplementary file 1 [file ao5c08479_si_001.pdf]

Supporting Information: Optoelectronic and excitonic study of XI_2 (X = Si, Ge, Sn, and Pb) monolayers envisaging potential technological applications

Bill Darwin Aparicio-Huacarpuma,[*,†,‡] José Artigas dos Santos Laranjeira,[¶] Kleuton Antunes Lopes Lima,[§] Elie Albert Moujaes,[‖] Alysson Martins Almeida Silva,[⊥] Julio Ricardo Sambrano,[¶] Alexandre Cavalheiro Dias,[*,#] and Luiz Antônio Ribeiro Júnior[*,†,‡]

†*Institute of Physics, University of Brasília, 70919-970, Brasília, DF, Brazil.*

‡*Computational Materials Laboratory, LCCMat, Institute of Physics, University of Brasília, 70919-970, Brasília, DF, Brazil.*

¶*Modeling and Molecular Simulation Group, São Paulo State University (UNESP), School of Sciences, Bauru, 17033-360, SP, Brazil.*

§*Department of Applied Physics and Center for Computational Engineering and Sciences, State University of Campinas, Campinas, SP, 13083-859, Brazil*

‖*Institute of Physics, Federal University of Bahia, Campus Ondina, 40170-115 Salvador, Brazil.*

⊥*University of Brasília, College of Technology, Department of Mechanical Engineering, 70910-900, Brasília, Brazil*

#*Institute of Physics and International Center of Physics, University of Brasília, 70919-970, Brasília, DF, Brazil.*

E-mail: bdaparicioh@gmail.com; alexandre.dias@unb.br; ribeirojr@unb.br

Contents

S1 Optimized POSCARs **S-2**

S1.1 SiI_2 . . . S-2

S1.2 GeI_2 . . . S-3

S1.3 SnI_2 . . . S-3

S1.4 PbI_2 . . . S-4

S2 Phonons and Thermodynamic Properties **S-4**

S3 Molecular Dynamics Simulations **S-7**

S4 Excitonic and Optical Properties **S-11**

S4.1 BSE simulation parameters . . . S-15

S5 Power Conversion Efficiency Mathematical Formalism **S-15**

S5.1 Shockley–Queisser Limit . . . S-17

S5.2 Spectroscopy Limited Maximum Efficiency . . . S-17

S6 Insights of Solar Harvesting Efficiency **S-18**

References **S-20**

S1 Optimized POSCARs

S1.1 SiI_2

```
"Si1 I2"
1.0000000000000000
4.1763640514713520 0.0000000000000000 0.0000000000000000
-2.0881820257356760 3.6168373640159377 0.0000000000000000
0.0000000000000000 0.0000000000000000 20.0000000000000000
Si I
```

```
1 2
Direct
0.0000000000000000 0.0000000000000000 0.5000000000000000
0.6666666670000012 0.3333333329999988 0.5855961465829793
0.3333333329999988 0.6666666670000012 0.4144038534170207
```

S1.2 GeI_2

```
"Ge1 I2"
1.0000000000000000
4.2422238880617495 0.0000000000000000 0.0000000000000000
-2.1211119440308748 3.6738736555917018 0.0000000000000000
0.0000000000000000 0.0000000000000000 20.0000000000000000
Ge I
1 2
Direct
0.0000000000000000 0.0000000000000000 0.5000000000000000
0.6666666670000012 0.3333333329999988 0.5885106979909480
0.3333333329999988 0.6666666670000012 0.4114893020090520
```

S1.3 SnI_2

```
"Sn1 I2"
1.0000000000000000
4.5435205297993102 0.0000000000000000 0.0000000000000000
-2.2717602648996551 3.9348042014229674 0.0000000000000000
0.0000000000000000 0.0000000000000000 20.0000000000000000
Sn I
1 2
Direct
0.0000000000000000 0.0000000000000000 0.5000000000000000
```

```
0.6666666670000012 0.3333333329999988 0.5930174678967077
0.3333333329999988 0.6666666670000012 0.4069825321032852
```

S1.4 PbI_2

```
"Pb1 I2"
1.0000000000000000
4.6398762822214739 0.0000000000000000 0.0000000000000000
-2.3199381411107369 4.0182507308465105 0.0000000000000000
0.0000000000000000 0.0000000000000000 20.0000000000000000
Pb I
1 2
Direct
0.0000000000000000 0.0000000000000000 0.5000000000000000
0.6666666670000012 0.3333333329999988 0.5936936873668301
0.3333333329999988 0.6666666670000012 0.4063063126331699
```

S2 Phonons and Thermodynamic Properties

Figures S1(a), S2(a), S3(a), and S4(a) shown positive frequencies in the phonon dispersion plots demonstrating its stability.

As shown in Figures S1 to S4, the Helmholtz free energy indicates that all four monolayers exhibit favorable synthesis conditions at temperatures above 100 K, suggesting that these systems can be synthesized under standard laboratory conditions, including room temperature (300 K). This observation is consistent with the thermodynamic trends in entropy and heat capacity (C_v), as presented in Figures S1(b), S2(b), S3(b) and S4(b), respectively.

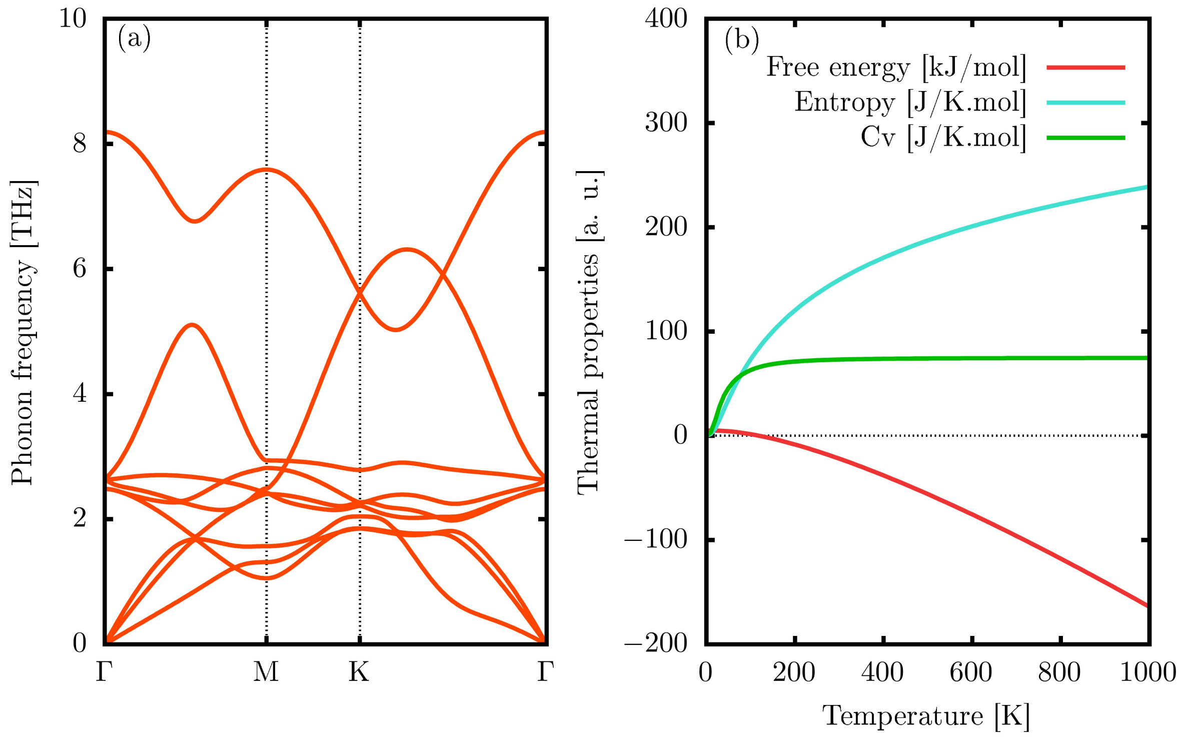

Figure S1: (a)Phonons and (b) Helmholtz free energy, Entropy and Heat capacity (Cv) for the SiI_2 monolayer as a function of the temperature (K), using the PBE functional.

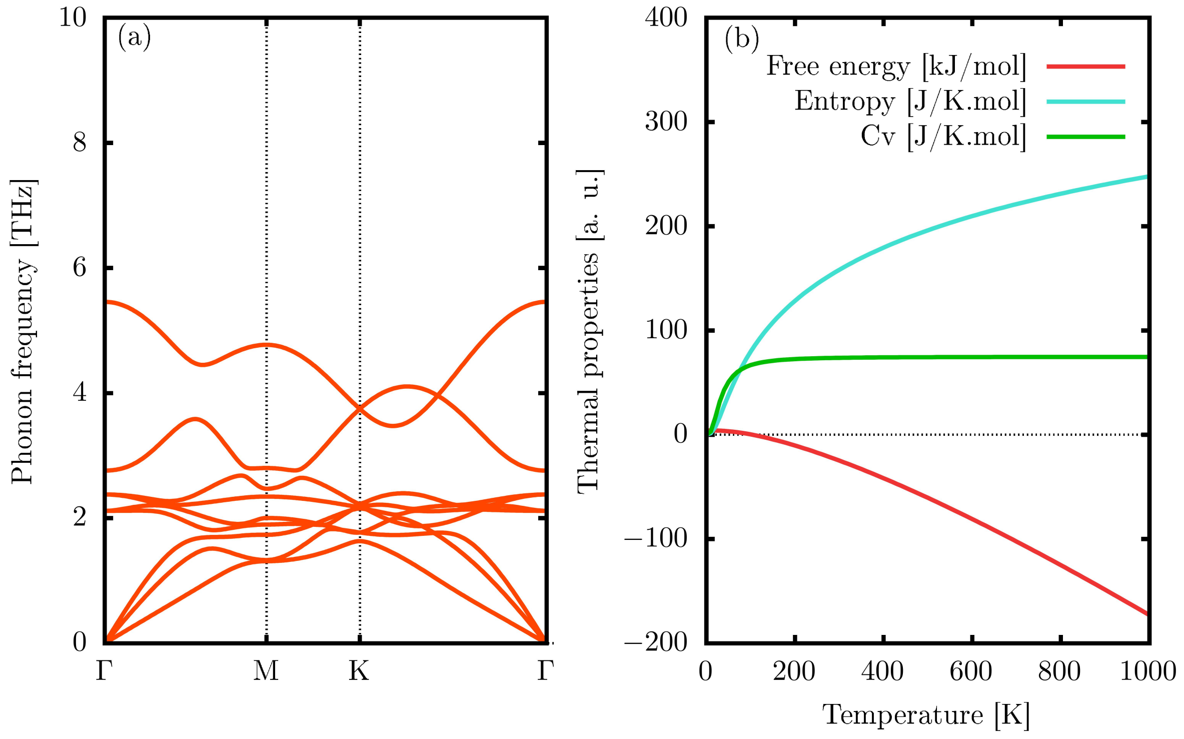

Figure S2: (a)Phonons and (b) Helmholtz free energy, Entropy and Heat capacity (Cv) for the GeI_2 monolayer as a function of the temperature (K), using the PBE functional.

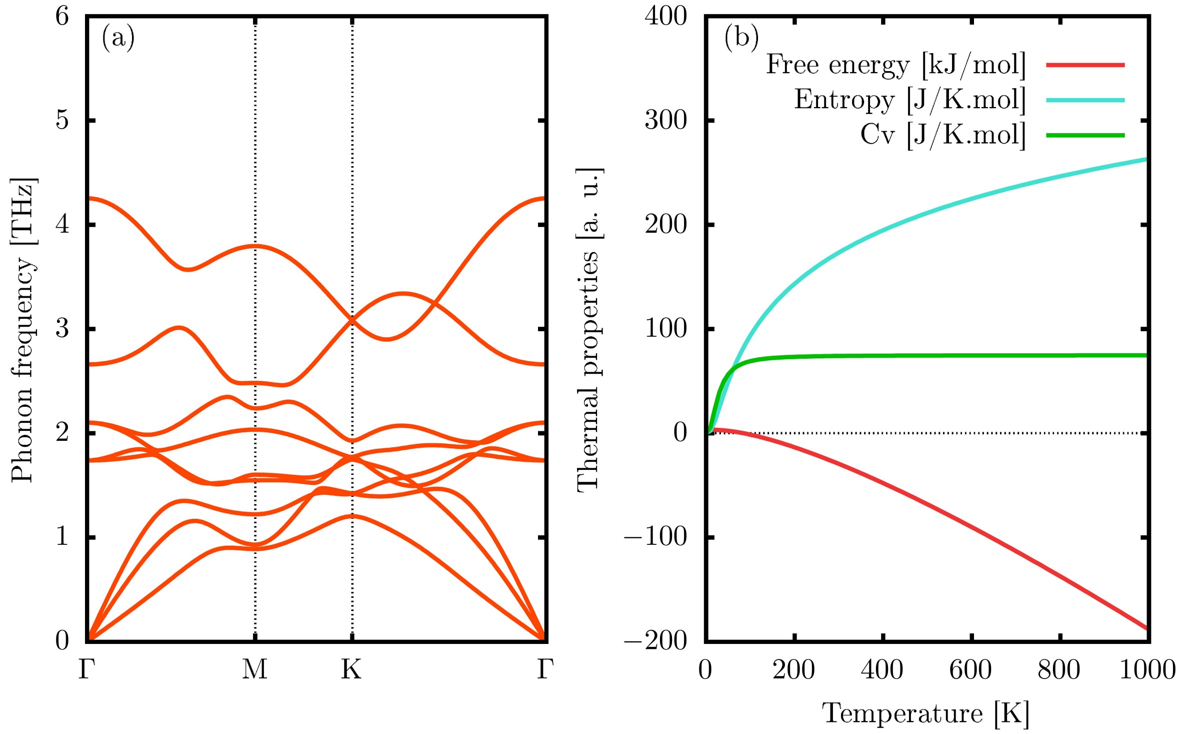

Figure S3: (a)Phonons and (b) Helmholtz free energy, Entropy and Heat capacity (Cv) for the SnI_2 monolayer as a function of the temperature (K), using the PBE functional.

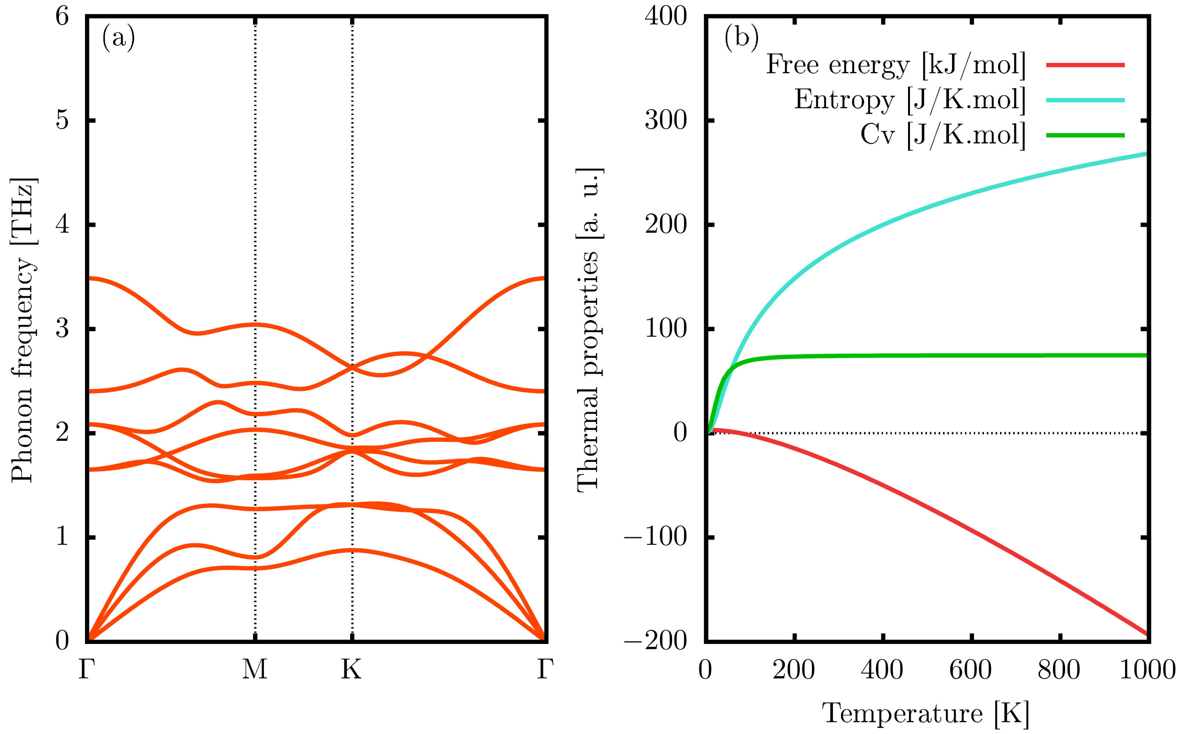

Figure S4: (a)Phonons and (b) Helmholtz free energy, Entropy and Heat capacity (Cv) for the PbI_2 monolayer as a function of the temperature (K), using the PBE functional.

S3 Molecular Dynamics Simulations

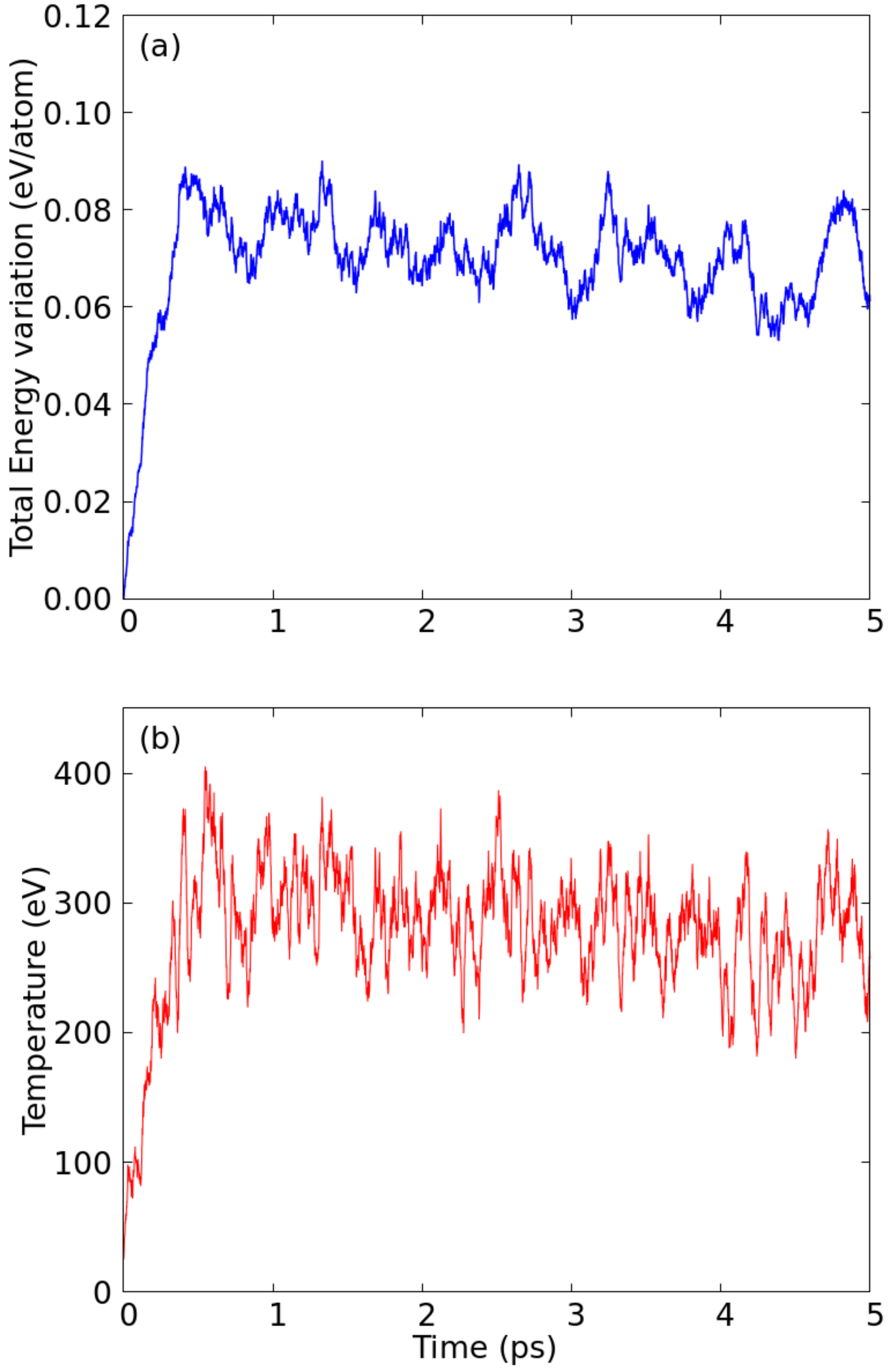

Figure S5: AIMD simulation for the SiI_2 monolayer, (a) total energy variation per atom and (b) temperature.

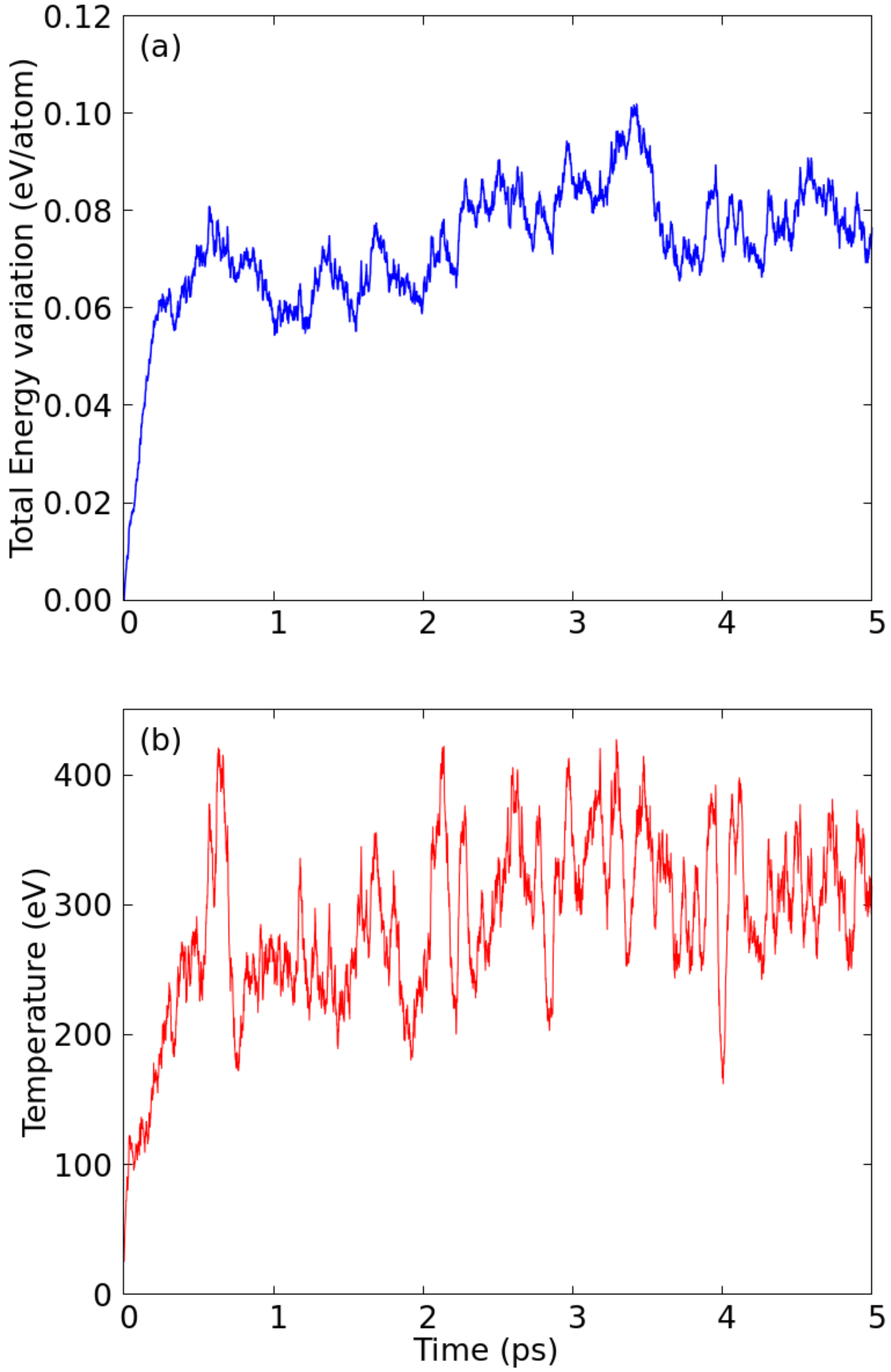

Figure S6: AIMD simulation for the GeI_2 monolayer, (a) total energy variation per atom and (b) temperature.

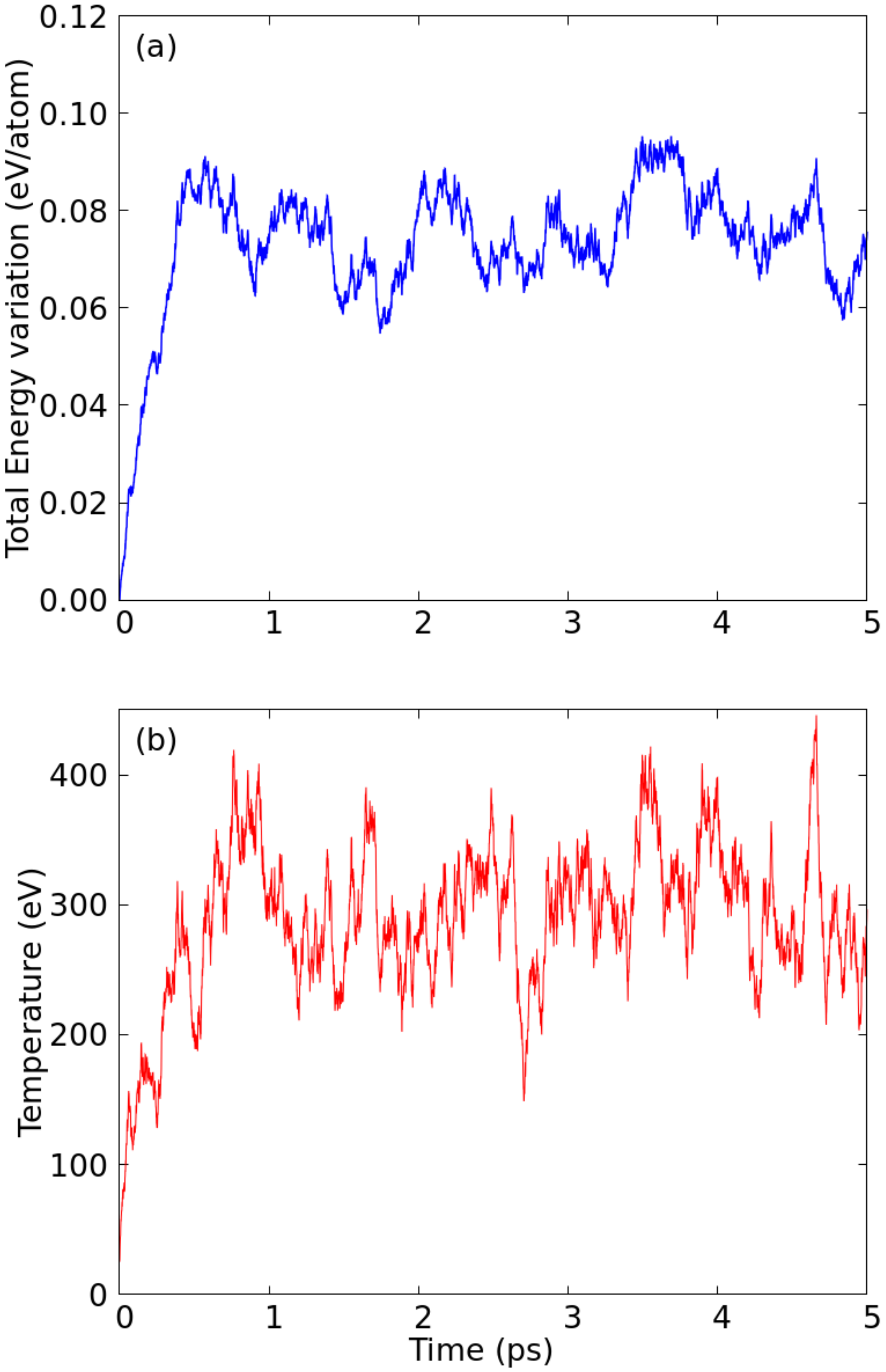

Figure S7: AIMD simulation for the SnI_2 monolayer, (a) total energy variation per atom and (b) temperature.

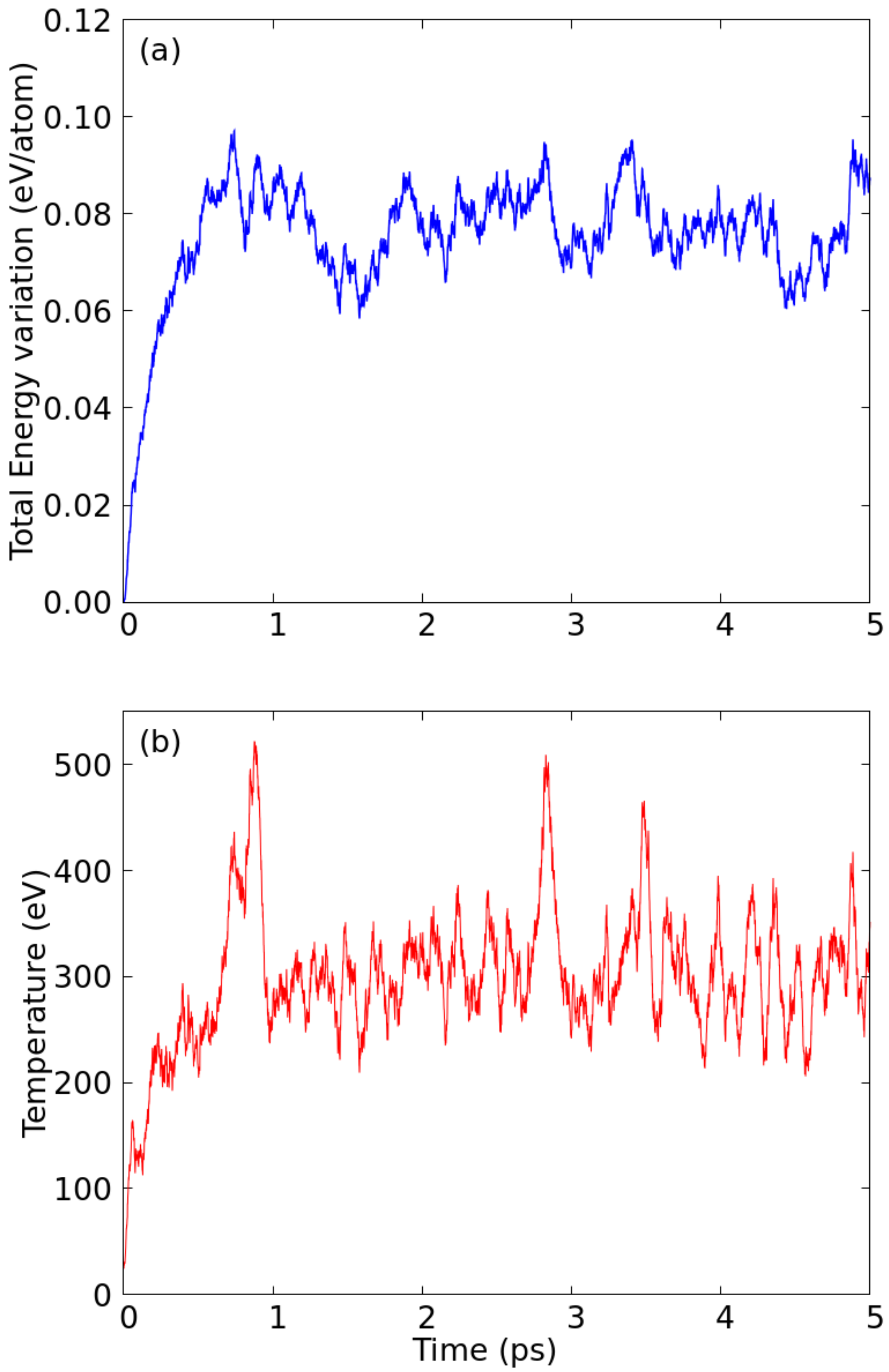

Figure S8: AIMD simulation for the PbI_2 monolayer, (a) total energy variation per atom and (b) temperature.

S4 Excitonic and Optical Properties

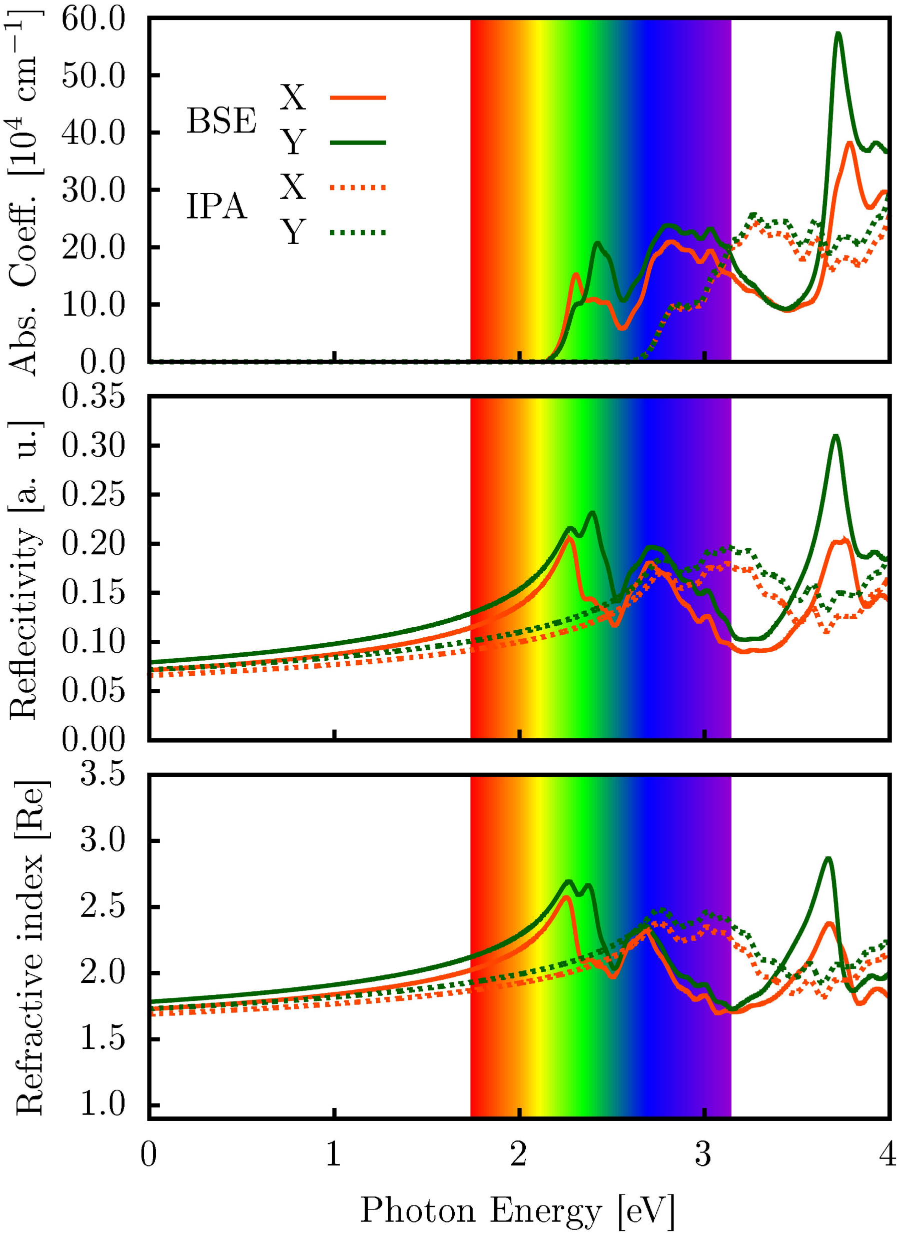

Figure S9: Absorption coefficient spectrum (upper panel), reflectibility (mid panel) and refractive index (lower panel) for SiI_2, at BSE (solid curves) and IPA (dashed curves) levels, considering linear light polarization at $\hat{x}$ (orange curves) and $\hat{y}$ (green curves) directions.

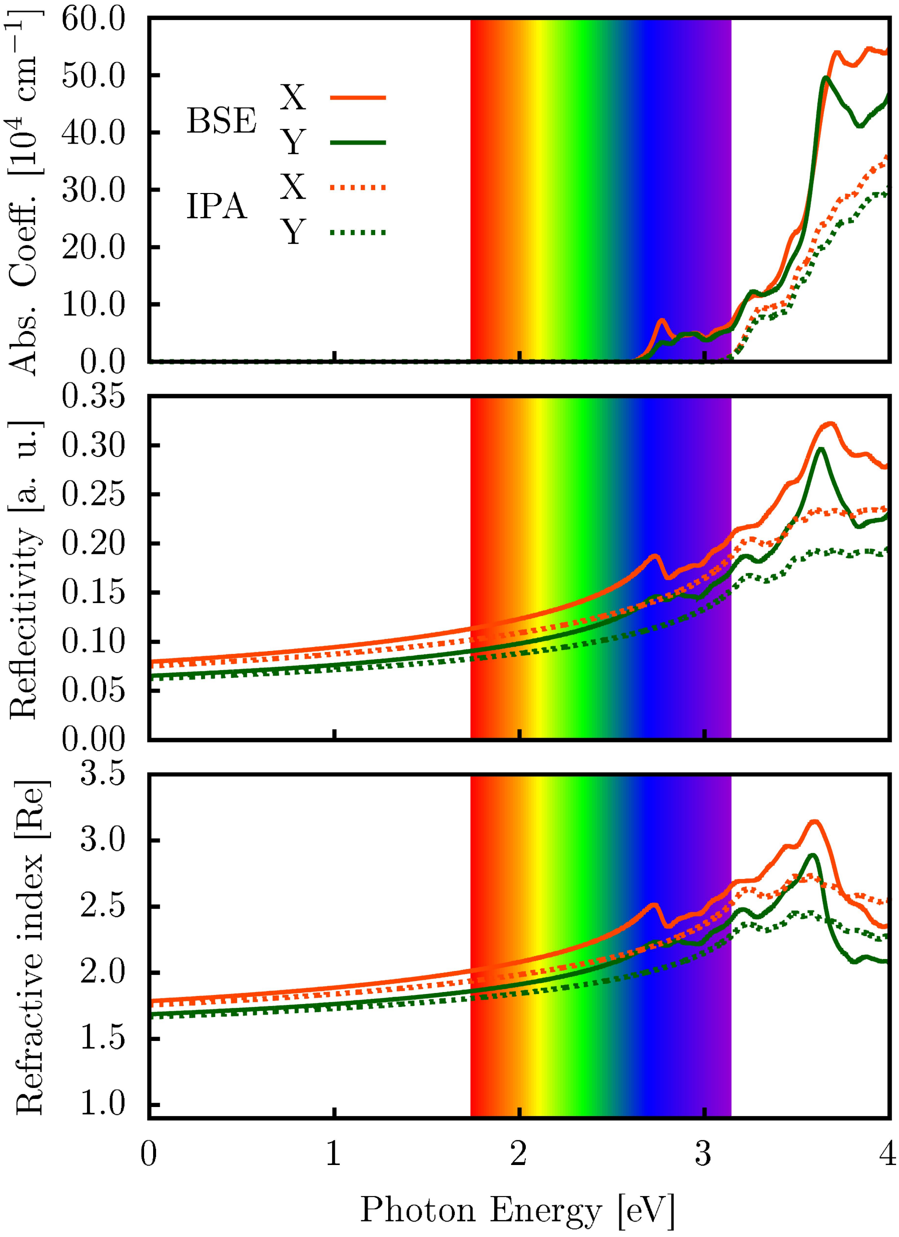

Figure S10: Absorption coefficient spectrum (upper panel), reflectibility (mid panel) and refractive index (lower panel) for GeI_2, at BSE (solid curves) and IPA (dashed curves) levels, considering linear light polarization at $\hat{x}$ (orange curves) and $\hat{y}$ (green curves) directions.

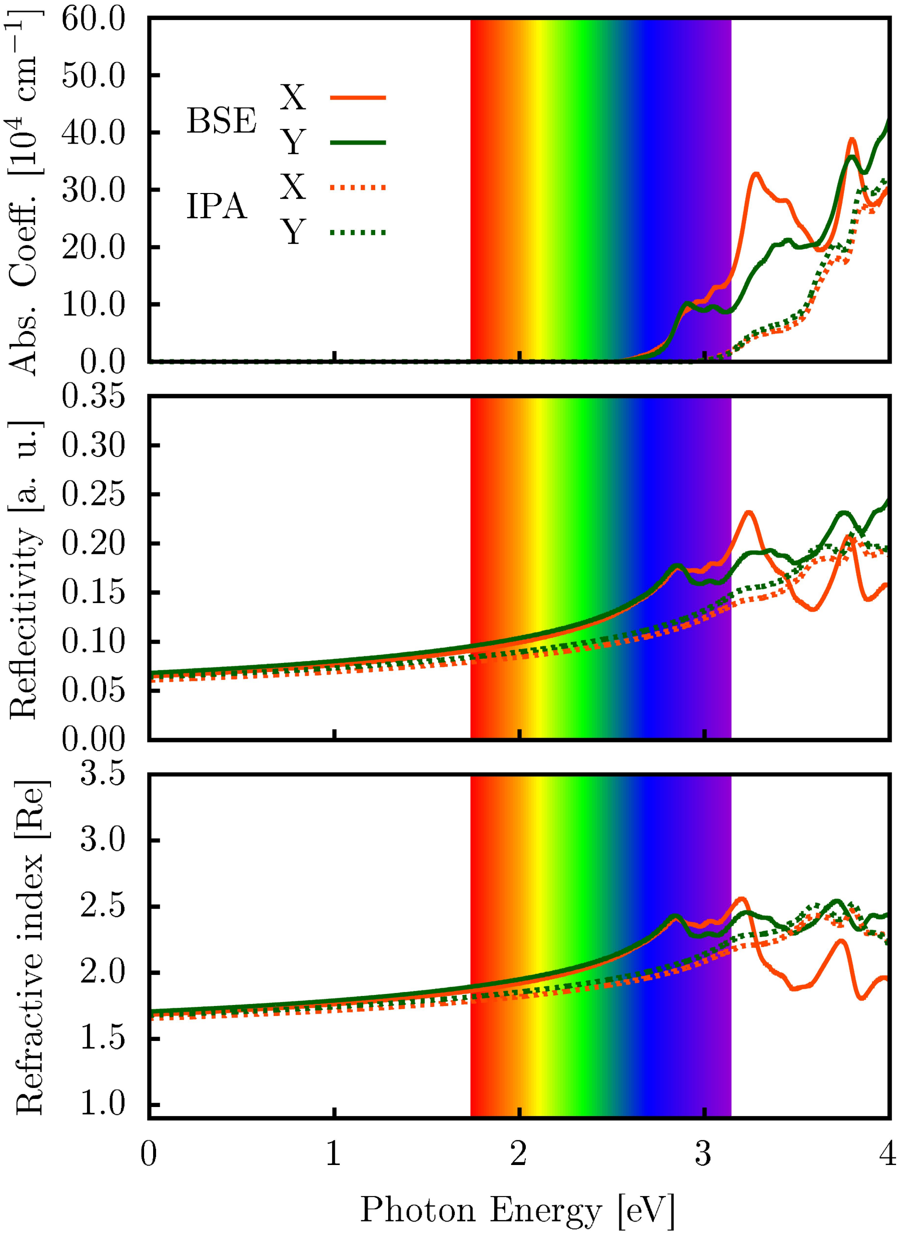

Figure S11: Absorption coefficient spectrum (upper panel), reflectibility (mid panel) and refractive index (lower panel) for SnI_2, at BSE (solid curves) and IPA (dashed curves) levels, considering linear light polarization at $\hat{x}$ (orange curves) and $\hat{y}$ (green curves) directions.

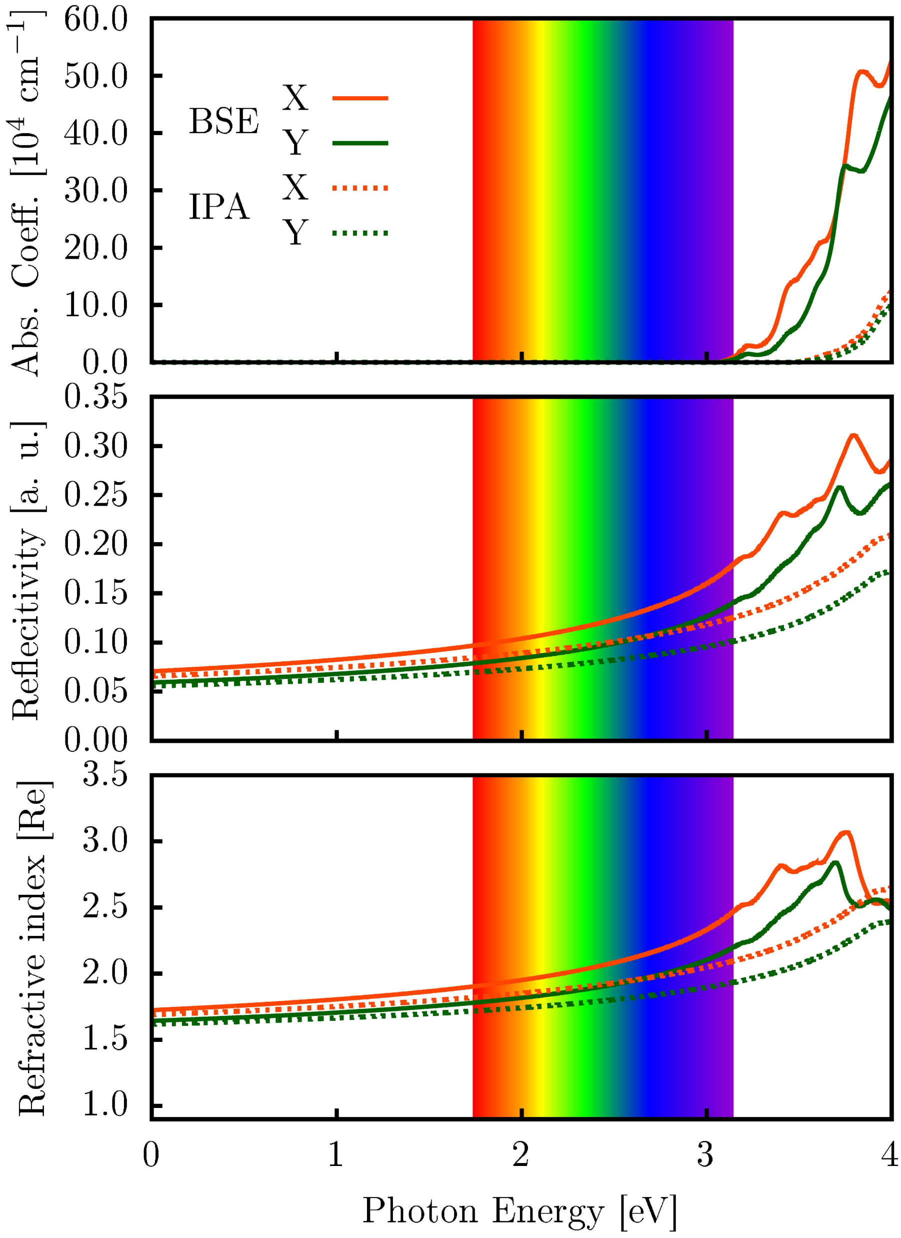

Figure S12: Absorption coefficient spectrum (upper panel), reflectibility (mid panel) and refractive index (lower panel) for PbI_2, at BSE (solid curves) and IPA (dashed curves) levels, considering linear light polarization at $\hat{x}$ (orange curves) and $\hat{y}$ (green curves) directions.

S4.1 BSE simulation parameters

Table S1: Parameters used for BSE simulations: **k**-points density, R_k ($\mathring{A}^{-1}$), and their correspondent **k**-mesh, n_v, number of valence bands, n_c, number of conduction bands and dielectric function smearing η (eV).

System	R_k	**k**-mesh	n_c	n_v	η
SiI_2	120	$33 \times 33 \times 1$	3	5	0.05
GeI_2	120	$33 \times 33 \times 1$	3	5	0.05
SnI_2	120	$30 \times 30 \times 1$	3	5	0.05
PbI_2	120	$30 \times 30 \times 1$	3	5	0.05

S5 Power Conversion Efficiency Mathematical Formalism

In this work, we employed two approaches to estimate the power conversion efficiency (PCE): the Shockley–Queisser (SQ) limit[S1], which offers a simplified yet idealized estimate, and the spectroscopy-limited maximum efficiency (SLME) scheme[S2], which incorporates material-specific optical properties. Both methods are grounded in the principle of detailed balance between photon absorption and emission in photovoltaic devices.

Below, we summarize the mathematical formalism that underpins these two approaches, following the treatment presented in Ref.[S3]. The PCE is defined as the ratio between the maximum electrical power density output (P_{PV}) and the total incident power density from the solar spectrum (P_{solar}), and is given by:

$$\mathrm{PCE} = \frac{P_{\mathrm{PV}}}{P_{solar}} , \tag{S1}$$

where

$$P_{solar} = \int_0^{\infty} P(E)dE . \tag{S2}$$

$P(E)$ is the global AMG1.5G[S4] solar energy flux, which is the standard solar spectrum for non-concentrated photovoltaic conversion, taking light absorption and scattering in the atmosphere into account. The output power density is described by the product $J(V)V$, as the

maximum output power density (P_{PV}) is obtained maximizing the $J-V$ characteristic of an illuminated solar cell:

$$P_{\text{PV}} = J(V_{max})V_{max} \ , \tag{S3}$$

where V_{max} is the voltage that results in the maximum output power density. In this method, the current density, $J(V)$, are described by the following expression:

$$J(V) = J_{sc} - \frac{J_0}{fr}\left(exp\left(\frac{eV}{k_{\text{B}}T}\right) - 1\right) \ , \tag{S4}$$

where k_{B} is Boltzmann's constant, e is the elementary charge, fr is the radiative electron-hole recombination fraction and T is the temperature of the solar cell. J_{sc} is the short-circuit current density, also known as the illuminated current or photogenerated current, calculated from the following expression,

$$J_{sc} = e\int_0^{\infty} a(E)\frac{P(E)}{E}dE \ . \tag{S5}$$

$a(E)$ is the absorbance, which is defined as the ratio of power absorbed by the solar device to the power of incident sunlight.

The reverse saturation current density (J_0) is calculated using the detailed balance principle under thermal equilibrium, where the rate of photon emission due to radiative recombination equals the rate of photon absorption from the environment. This condition assumes the solar cell is coupled to an ideal heat sink such that its temperature matches the ambient temperature. Consequently, the surrounding environment is modeled as a black body radiator at temperature T as

$$J_0 = e\pi\int_0^{\infty} a(E)\Phi_{bb}(E)dE \ , \tag{S6}$$

where,

$$\Phi_{bb}(E) = \frac{2E^2}{h^3v_c^2}\left(e^{\frac{E}{k_{\text{B}}T}} - 1\right)^{-1} \ , \tag{S7}$$

and h is Planck's constant and v_c is the speed of light.

S5.1 Shockley–Queisser Limit

The absorbance $a(E)$ is modeled as a Heaviside step function in the SQ-limit approximation. This feature means that all photons with energy greater than or equal to the band gap energy (E_g) are fully absorbed, i.e., $a(E) = 1$ for $E \geq E_g$ and $a(E) = 0$ for $E < E_g$. Additionally, this model assumes a radiative recombination fraction $fr = 1$, meaning that radiative recombination is the only loss mechanism and non-radiative processes such as Auger recombination are neglected—even in the case of indirect band gap materials[S5,S6].

In this work, the PCE values obtained using the SQ-limit were taken from Rühle's compilation[S7], which considers a solar cell operating at 298.15 K, with radiative emission occurring from both the front and rear surfaces of the device. Under this approximation, the only input required from DFT is the fundamental energy band gap, regardless of whether it is direct or indirect. Excitonic effects can also be incorporated into the SQ-limit framework by replacing the electronic band gap with the optical band gap, defined by the bright excitonic ground-state energy.

S5.2 Spectroscopy Limited Maximum Efficiency

In contrast to the SQ-limit approach, the SLME approximation requires the total absorption coefficient $A(\omega)$—defined as $A_{xx}(\omega) + A_{yy}(\omega)$—along with the material thickness Δ and the electronic and optical band gaps. This method also accounts for non-radiative recombination by introducing a radiative recombination fraction fr, modeled using a Boltzmann factor[S2]:

$$fr = e^{-\frac{\delta}{k_B T}} , \tag{S8}$$

where $\delta = E_{op} - E_g$, with E_{op} representing the optical band gap and E_g the fundamental (direct or indirect) electronic band gap.

The absorbance $a(E)$ is evaluated under the same assumptions as the SQ-limit, using the expression[S2,S8]:

$$a(E) = 1 - e^{-2A(\omega)\Delta} \, , \tag{S9}$$

where $E = \hbar\omega$. Excitonic effects are incorporated both through the total absorption coefficient and in the δ parameter, where the optical band gap E_{op} is replaced by the bright exciton ground-state energy E_X^{br}. In this study, all calculations were performed at a temperature of 298.15 K.

The SLME_{max} values reported in this work were obtained using the expressions above. However, in this case, the absorbance was approximated by a Heaviside step function, as in the SQ limit. The key distinction lies in the inclusion of the recombination fraction f_r, which differentiates the behavior of direct and indirect band gap semiconductors.

S6 Insights of Solar Harvesting Efficiency

Table S2: Maximum achieved PCE at the BSE level, short circuit current density J_{sc} (W/Vm^2), open circuit voltage, V_{oc} (V), recombination fraction, fr, fill factor, FF(%), monolayer thickness plus vdW length, t (nm), PCS obtained by SLME (PCE^{SLME}) (%), PCE obtained by SLME considering 100 % of photon absorbance starting from the optical band gap (PCE^{SLME}_{max})(%), and power conversion efficiency obtained in the Shockley-Queisser limit considering the gap band gap (PCE^{SQ})(%), at room temperature T=300 K.

System	J_{sc}	V_{oc}	$-ln(fr)$	FF	t	PCE^{SLME}	PCE^{SLME}_{max}	PCE^{SQ}
SiI_2	3.59	1.64	12.15	91.99	0.66	0.54	13.57	16.37
GeI_2	0.96	2.10	11.72	93.43	0.68	0.19	6.61	7.64
SnI_2	1.24	1.99	13.96	93.13	0.69	0.23	7.83	9.39
PbI_2	0.35	2.49	13.81	94.27	0.70	0.08	2.35	2.71

Table S3: Maximum achieved PCE at the IPA level, short circuit current density J_{sc} (W/Vm^2), open circuit voltage, V_{oc} (V), recombination fraction, fr, fill factor, FF(%), monolayer thickness plus vdW length, t (nm), PCE obtained by SLME (PCE^{SLME}) (%), PCE by SLME considering 100 % of phonon absorbance starting from the optical band gap (PCE^{SLME}_{max})(%), and PCE obtained in the Shockley-Queisser limit considering the gap band gap (PCE^{SQ})(%), at room temperature T=300 K.

System	J_{sc}	V_{oc}	$-ln(fr)$	FF	t	PCE^{SLME}	PCE^{SLME}_{max}	PCE^{SQ}
SiI_2	1.30	1.99	15.43	93.14	0.66	0.24	6.45	7.84
GeI_2	0.38	2.44	15.32	94.17	0.68	0.09	2.24	2.63
SnI_2	0.29	2.36	14.46	94.01	0.69	0.06	3.02	3.54
PbI_2	0.02	2.85	13.31	94.87	0.70	0.01	0.72	0.81

References

(S1) Shockley, W.; Queisser, H. J. Detailed Balance Limit of Efficiency of p-n Junction Solar Cells. *J. Appl. Phys.* **1961**, *32*, 510–519, DOI: 10.1063/1.1736034.

(S2) Yu, L.; Zunger, A. Identification of Potential Photovoltaic Absorbers Based on First-Principles Spectroscopic Screening of Materials. *Phys. Rev. Lett.* **2012**, *108*, 068701, DOI: 10.1103/PhysRevLett.108.068701.

(S3) Bercx, M.; Saniz, R.; Partoens, B.; Lamoen, D. Exceeding the Shockley–Queisser Limit Within the Detailed Balance Framework. In *Many-body Approaches at Different Scales: A Tribute to Norman H. March on the Occasion of his 90th Birthday*; Angilella, G., Amovilli, C., Eds.; Springer International Publishing: Cham, 2018; pp 177–184, DOI: 10.1007/978-3-319-72374-7_15.

(S4) ASTM-G173-03 Standard Tables for Reference Solar Spectral Irradiances: Direct Normal and Hemispherical on 37° Tilted Surface, ASTM International, West Conshohocken, PA (2012). 2012; https://doi.org/10.1520/g0173-03r20.

(S5) Huldt, L. Band-to-band auger recombination in indirect gap semiconductors. *Physica Status Solidi (a)* **1971**, *8*, 173–187, DOI: 10.1002/pssa.2210080118.

(S6) Green, M. Limits on the open-circuit voltage and efficiency of silicon solar cells imposed by intrinsic Auger processes. *IEEE Transactions on Electron Devices* **1984**, *31*, 671–678, DOI: 10.1109/t-ed.1984.21588.

(S7) Rühle, S. Tabulated values of the Shockley–Queisser limit for single junction solar cells. *SolEn* **2016**, *130*, 139–147, DOI: https://doi.org/10.1016/j.solener.2016.02.015.

(S8) Duan, J.; Xu, H.; Sha, W. E. I.; Zhao, Y.; Wang, Y.; Yang, X.; Tang, Q. Inorganic perovskite solar cells: an emerging member of the photovoltaic community. *Journal of Materials Chemistry A* **2019**, *7*, 21036–21068, DOI: 10.1039/c9ta06674h.
